# LyeTx I mnΔKL, a New Synthetic Peptide Derived from a *Lycosa erythrognatha* Toxin, with Potent In Vitro and In Vivo Activity Against Methicillin-Resistant *Staphylococcus aureus*

**DOI:** 10.3390/toxins18080323

**Published:** 2026-07-25

**Authors:** Waleska Stephanie da Cruz Nizer, Giulliana Altaf dos Santos, William Gustavo Lima, Felipe Henrique de Souza Silva, Wanderson Aparecido Brandão Candido, Amanda Neves de Souza, Giovanna Paula Araújo, Rodrigo Moreira Verly, Maria Elena de Lima

**Affiliations:** 1 Faculdade de Saúde Santa Casa de Belo Horizonte, Programa de Pós-Graduação Stricto Sensu de Medicina e Biomedicina, Belo Horizonte 30260-070, MG, Brazil; giullialtaf@gmail.com (G.A.d.S.); williamlimainfecto@gmail.com (W.G.L.); felipehssilva@gmail.com (F.H.d.S.S.); 2 Departamento de Química, Instituto de Ciências Exatas, Universidade Federal dos Vales do Jequitinhonha e Mucuri, Diamantina 39100-000, MG, Brazil; wanderson.aparecido@ufvjm.edu.br (W.A.B.C.); amandaneves.200@gmail.com (A.N.d.S.); giovanna.araujo@ufvjm.edu.br (G.P.A.); verly.rodrigo@ufvjm.edu.br (R.M.V.)

**Keywords:** antimicrobial peptides, methicillin-resistant *Staphylococcus aureus*, skin infections, Gram-positive bacteria, drug development, LyeTx I mnΔKL

## Abstract

The emergence of multidrug-resistant (MDR) bacteria, particularly methicillin-resistant *Staphylococcus aureus* (MRSA), represents a major global health challenge by limiting the current therapeutic options. In this context, antimicrobial peptides (AMPs) have been widely studied for their potent antimicrobial properties. In this study, we evaluated the anti-MRSA effect of a novel AMP, LyeTx I mnΔKL, derived from a toxin of *Lycosa erythrognatha*. Its activity was evaluated in vitro by minimal inhibitory and bactericidal concentrations (MIC and MBC), antibiofilm effect, membrane interaction, cytotoxicity, synergistic interaction with vancomycin, and in vivo in an MRSA murine wound/abscess infection model. LyeTx I mnΔKL showed enhanced antimicrobial activity against clinical MRSA isolates compared to its prototype (LyeTx I mnΔK), with MIC_50_ and MBC_50_ of 2 and 8 µM and 16 and 32 µM, respectively. Furthermore, LyeTx I mnΔKL exhibited a rapid bactericidal effect and a pronounced ability to inhibit biofilm formation and disrupt mature biofilms. LyeTx I mnΔKL interacts with bacterial membranes, adopts an α-helical structure, and induces membrane disruption and leakage of intracellular material. In vivo, topical treatment with LyeTx I mnΔKL reduced MRSA burden compared with LyeTx I mnΔK and untreated controls (log_10_ CFU/g of wound of 2.4, 4.4, and 6.5 for LyeTx I mnΔKL, LyeTx I mnΔK, and the saline group, respectively). However, increased cytotoxicity remains a significant limitation. Overall, LyeTx I mnΔKL is a promising anti-MRSA candidate for topical use with potent antibiofilm and in vivo activity.

## 1. Introduction

The rise of multidrug-resistant (MDR) bacteria has created a global public health crisis. According to a recent Antimicrobial Resistance Collaborators review, it is estimated that 1.91 million (confidence interval (CI) 1.56–2.26) deaths attributable to antimicrobial resistance (AMR) and 8.22 million (CI 6.85–9.65) deaths associated with AMR could occur globally in 2050 [1]. Among the pathogens of concern, *Staphylococcus aureus* is particularly relevant due to its prevalence in community and hospital settings [2]. This Gram-positive pathogen causes a wide variety of infections, including bacteremia, pneumoniae, endocarditis, and skin, soft tissue, and device-related infections [3]. In particular, methicillin-resistant *S. aureus* (MRSA) is frequently associated with outbreaks in healthcare environments [4,5]. Its high pathogenicity is attributed to the increased resistance to methicillin and several other β-lactam antibiotics [6] and the ability to form biofilms [7,8]. For instance, the Centers for Disease Control and Prevention (CDC) reported a 13% increase in the incidence of hospital-acquired MRSA infections in the USA during the COVID-19 pandemic [9]. Importantly, the increased resistance of MRSA has limited the use of current therapeutic options, which are limited to a few antibiotics, including vancomycin, mupirocin, daptomycin, and linezolid [10,11]. However, the use of these agents is restricted by treatment failure, high toxicity, slow bactericidal action, and low tissue penetration [12,13,14].

Antimicrobial peptides (AMPs) have been widely explored as promising antimicrobial agents against MDR bacteria. AMPs are small molecules produced by a wide variety of organisms, including amphibians, arthropods, plants, humans, and microorganisms [15]. In addition to their potent antimicrobial effects due to membrane interaction, AMPs also present antibiofilm [16,17] and immunomodulatory activities [18,19]. However, their clinical use is limited by factors such as high production costs, low stability, and elevated cytotoxicity [15]. To address these challenges, structural modifications, such as amino acid substitutions, insertions or deletions, cyclization, and conjugation to hydrophobic residues, have been commonly employed [20].

Arthropod venoms, such as those from spiders and bees, are important sources of bioactive molecules, including AMPs [21,22]. Our research group has focused on the isolation, biochemical characterization, synthesis, structural modification, and evaluation of the biological effects of peptides from arthropod venoms [23,24,25]. Among the molecules studied is LyeTx I (H-IWLTALKFLGKNLGKHLAKQQLAKL-NH_2_; Molecular weight: 2832.48 Da), isolated from the venom of the spider *Lycosa erythrognatha* [26], a species commonly found in Brazil and popularly known as “*aranha-de-grama*”, “*tarântula*”, “*aranha-de-jardim*” or “*aranha lobo*” [27]. LyeTx I presented a promising antimicrobial effect against *Escherichia coli*, *S. aureus, Candida krusei* (currently referred to as *Pichia kudriavzevii*), and *Cryptococcus neoformans*, probably by membrane disruption [26]. Follow-up studies focused on structural modifications of LyeTx I to improve its antimicrobial activity. In this vein, the removal of amino acid residues from the C-terminal region of LyeTx I and the insertion of a lysine at position 5 resulted in a short peptide of 16 amino acid residues called LyeTx I mnΔK (H-IWLTKALKFLGKNLGK-NH2; Molecular weight: 1829.28 Da; charge: +5) [28]. The antimicrobial effect of LyeTx I mnΔK has been shown against bacterial and fungal species, including *Acinetobacter baumannii* [23,28], MRSA [25], *E. coli*, *Pseudomonas aeruginosa*, *P. kudriavzevii*, *C. neoformans*, *C. gattii* [28], and *Candida albicans* [29].

Based on the promising activity of LyeTx I mnΔK and given that modifications to peptide structure can increase their antimicrobial properties, we designed a novel peptide by inserting a leucine residue (L) at the C-terminal region of LyeTx I mnΔK. This modification was chosen since leucine increases hydrophobic interactions with bacterial membranes, potentiating the antimicrobial activity of AMPs [30,31]. The novel synthetic peptide was called LyeTx I mnΔKL (H-IWLTKALKFLGKNLGKL-NH_2_; Molecular weight: 1942.44 Da; 17 amino acids; charge: +5). Therefore, this study aimed to evaluate the antibacterial activity of LyeTx I mnΔKL against MRSA, its interaction with bacterial membranes, cytotoxicity, antibiofilm activity, and synergism with vancomycin. In addition, we evaluated the interaction of this peptide with lipid bilayers by circular dichroism (CD) and isothermal titration calorimetry (ITC). Furthermore, we also evaluated the potential use of LyeTx I mnΔKL as a topical agent using an in vivo wound/abscess infection model. The insertion of an L on the structure of LyeTx I mnΔKL increased its anti-MRSA activity compared to the prototype LyeTx I mnΔK. Furthermore, LyeTx I mnΔKL presented a rapid bactericidal effect and led to a reduction of biofilm formation and mature biofilms. In vivo, LyeTx I mnΔKL presented a more pronounced effect in a wound/abscess murine model.

## 2. Results

### 2.1. LyeTx I mnΔKL Presents an Increased Antimicrobial Effect Against MRSA Clinical Isolates

The minimal inhibitory concentration (MIC) of LyeTx I mnΔKL against MRSA strains was determined by the broth microdilution method in Mueller–Hinton broth (MHB). As shown in Table 1, LyeTx I mnΔKL presented increased anti-MRSA activity compared to the peptide LyeTx I mnΔK. LyeTx I mnΔKL exhibited MIC values ranging from 2 to 4 µM, with MIC_50_ of 2 µM, while LyeTx I mnΔK displayed MIC values ranging from 4 to 32 µM, with MIC_50_ eightfold higher (16 µM). The positive control, vancomycin, showed an MIC_50_ of 2 µM, the same value as for LyeTx I mnΔKL.

Next, we determined the minimal bactericidal concentration (MBC) values of the AMPs (Table 1). LyeTx I mnΔKL showed overall lower MBC values (MBC_50_ of 8 µM) than LyeTx I mnΔK (MBC_50_ of 32 µM) and only two-fold higher than vancomycin (MBC_50_ of 4 µM). Overall, these results indicate that inserting a leucine residue into the LyeTx I mnΔK structure enhances its antibacterial potency against MRSA strains, reaching levels comparable to those of vancomycin.

### 2.2. LyeTx I mnΔKL Presents an Increased Killing Effect

**Figure 1:**
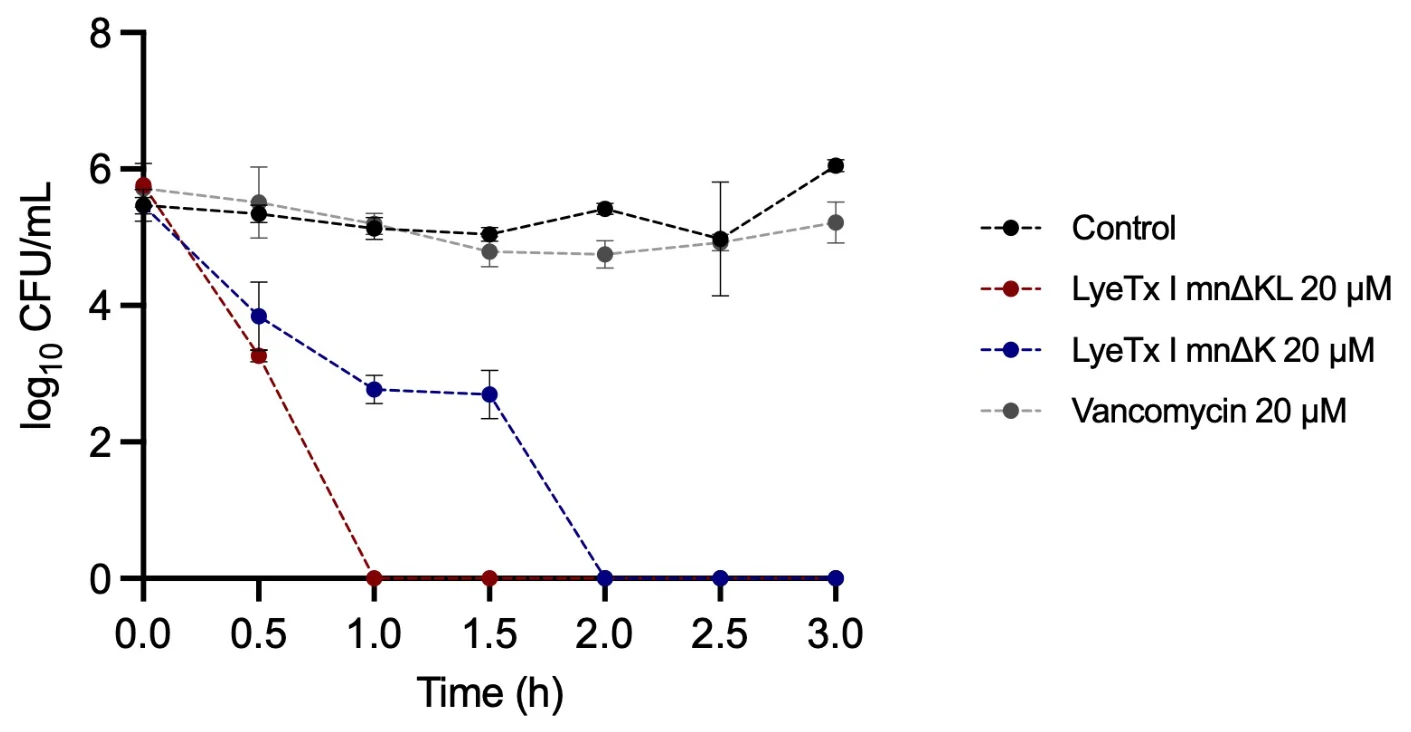
Time-kill kinetics of LyeTx I mnΔKL. MRSA USA 300 (10^6^ CFU/mL) was treated with 20 μM LyeTx I mnΔKL, LyeTx I mnΔK, or vancomycin for 3 h, serially diluted, and plated out on mannitol-salt agar.

To evaluate the killing kinetics of MRSA exposed to LyeTx I mnΔKL, a time-kill curve was performed. For this, MRSA USA 300 (10^6^ CFU/mL) was treated with LyeTx I mnΔKL at a lethal concentration of 20 µM for 3 h, and CFU was determined. As shown in Figure 1, LyeTx I mnΔKL presented a rapid killing effect against MRSA cells, inducing an approximately 2-fold reduction in log_10_ CFU/mL (from 5.76 to 3.26) after 30 min and complete bacterial eradication after 1 h. On the other hand, LyeTx I mnΔK reduced bacterial load from 5.5 to 2.8 in the first h, with complete elimination of bacterial cells after 2 h. Vancomycin, used as a positive control, did not show bactericidal activity under the time of time tested (3 h). Analysis of the area under the curve revealed significant differences (*p* < 0.0001) between the peptide-treated groups and the untreated control (15.8 ± 0.39 for the untreated control, 6.02 ± 0.04 for LyeTx I mnΔK, 3.072 ± 0.12 for LyeTx I mnΔKL, and 15.32 ± 0.26 for vancomycin), confirming a greater and more pronounced reduction in bacterial viability of LyeTx I mnΔKL compared to LyeTx I mnΔK and vancomycin.

### 2.3. LyeTx I mnΔKL Presents a Pronounced Effect Against MRSA Biofilms

**Figure 2:**
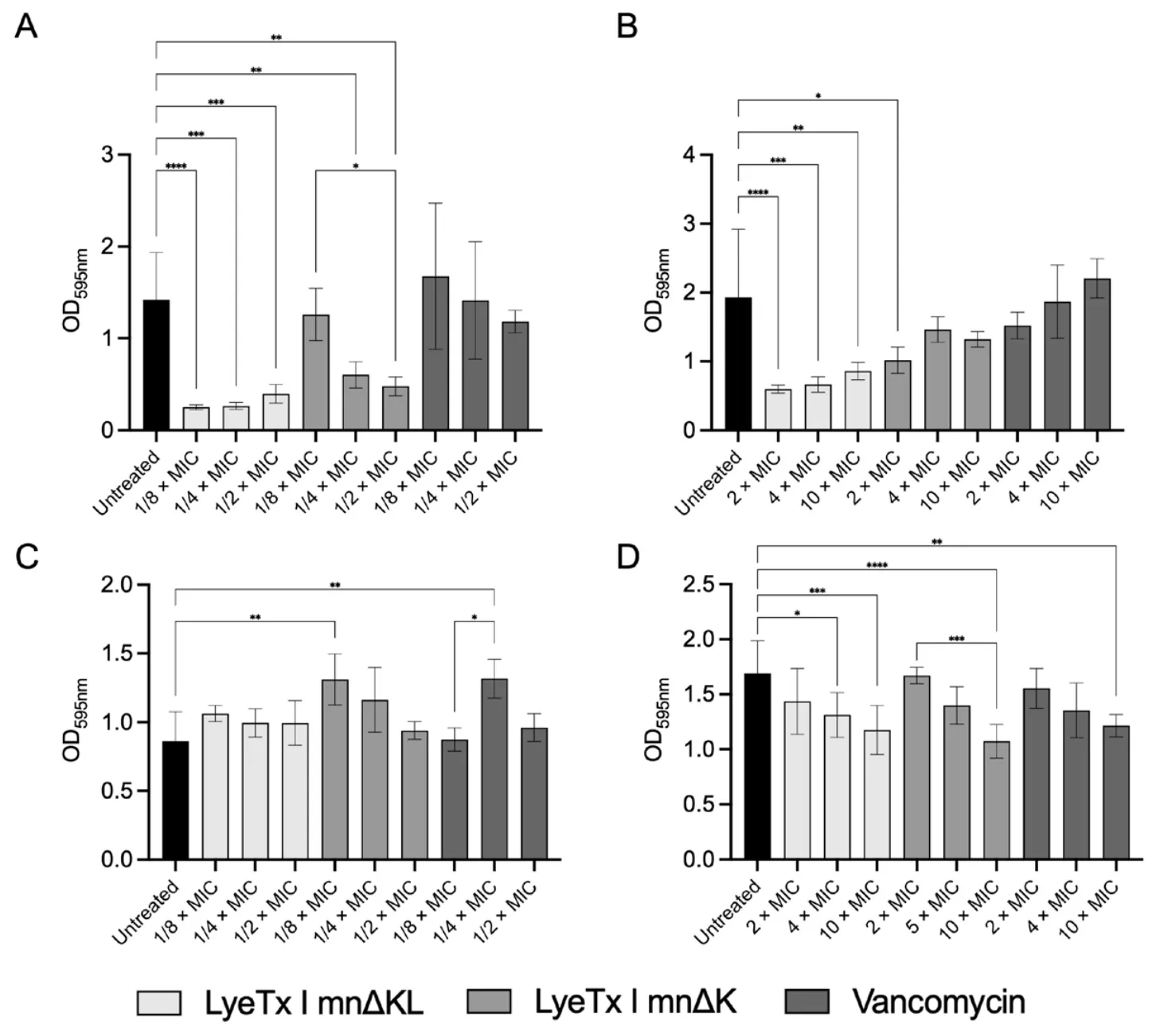
Effect of LyeTx I mnΔKL on MRSA biofilm formation and mature biofilms. (**A**) Effect of LyeTx I mnΔKL on biofilm formation and (**B**) mature biofilms assessed by the crystal violet assay. Cell viability of (**C**) biofilm formation and (**D**) mature biofilms assessed by the MTT assay. For biofilm formation assays, MRSA USA 300 cells (10^6^ CFU/mL) were diluted in MHB supplemented with 1% glucose and incubated with 1/8, ¼, or ½ × MIC of LyeTx I mnΔKL, LyeTx I mnΔK, or vancomycin in 96-well microtiter plates for 24 h at 37 °C under static conditions. For mature biofilm assays, MRSA USA 300 (10^6^ CFU/mL) in MHB with 1% glucose was incubated for 24 h at 37 °C to allow biofilm formation, followed by treatment with 2, 4, or 10 × MIC of LyeTx I mnΔKL, LyeTx I mnΔK, or vancomycin for 24 h at 37 °C. Biofilms were stained with 0.1% crystal violet solution or MTT, and the OD was measured at 595 nm using a microplate reader. * *p* < 0.05; ** *p* < 0.01; *** *p* < 0.001; **** *p* < 0.0001.

Biofilms are the predominant form of bacteria in nature and are responsible for the increased resistance of microbial pathogens [32,33]. We evaluated the effect of LyeTx I mnΔKL on biofilm formation and mature biofilms using the crystal violet assay and cell viability by the 3-(4,5-Dimethylthiazol-2-yl)-2,5-Diphenyltetrazolium Bromide (MTT) assay. For the effect on biofilm formation, MRSA cells (10^6^ CFU/mL) were incubated with sub-lethal concentrations (i.e., 1/8, ¼, and ½ × MIC) of LyeTx I mnΔKL, LyeTx I mnΔK, or vancomycin for 24 h. For the effect on mature biofilms, lethal concentrations (i.e., 2, 4, and 10 × MIC) of the compounds were added for 24 h to 24 h-grown biofilms. Biofilms were stained with 0.1% crystal violet for biofilm biomass quantification or with MTT for cell viability evaluation (Figure 2).

As shown in Figure 2A, LyeTx I mnΔKL showed a more pronounced effect on biofilm formation, with OD_595nm_ values of 0.25, 0.26, and 0.39 for 1/8, ¼, and ½ × MIC, respectively, compared to the untreated control (OD_595nm_ of 1.4). LyeTx I mnΔK significantly reduced biofilm at the highest concentrations (i.e., ¼ and ½ × MIC), with OD_595nm_ of 0.6 and 0.48, respectively. Vancomycin did not affect MRSA biofilm formation under our experimental conditions (Figure 2A). Similarly, LyeTx I mnΔKL exhibited a pronounced effect against 24 h MRSA biofilms at all concentrations tested (2, 4, and 10 × MIC), with OD_595nm_ values of 0.6, 0.66, and 0.9, respectively. In contrast, LyeTx I mnΔK reduced biofilm biomass only at the lowest concentration (2 × MIC; OD_595nm_ of 1.01) compared to the untreated control (OD_595nm_ of 1.9). Vancomycin showed no antibiofilm activity under our experimental conditions (Figure 2B).

The cell viability of biofilm cells treated with LyeTx I mnΔKL, LyeTx I mnΔK, or vancomycin, compared to the untreated control, was then assessed by the MTT reduction assay. LyeTx I mnΔKL did not affect MRSA cell viability during biofilm formation at the tested concentrations (1/8, ¼, and ½ × MIC), while an increase in cell viability was observed for LyeTx I mnΔK at 1/8 × MIC (OD_595nm_ of 1.3) and vancomycin at ¼ × MIC (OD_595nm_ of 1.32) compared to the untreated control (OD_595nm_ of 0.86) (Figure 2C). For mature biofilms, LyeTx I mnΔKL significantly reduced MRSA viability at 4 and 10 × MIC, with OD_595nm_ values of 1.3 and 1.2, respectively, compared to 1.7 in the untreated control. LyeTx I mnΔK and vancomycin significantly reduced cell viability only at 10 × MIC, with OD_595nm_ values of 1.1 and 1.2, respectively (Figure 2D). Overall, these results indicate that LyeTx I mnΔKL has a more pronounced effect on biofilms than LyeTx I mnΔK, reducing biofilm biomass and affecting the cell viability of mature biofilms only.

### 2.4. Effect of the Combination of LyeTx I mnΔKL with Vancomycin

Drug combinations represent an important strategy in antimicrobial therapy to enhance therapeutic efficacy and prevent the development of resistance [34]. Then, we evaluated the effect of the combination of LyeTx I mnΔKL with vancomycin using the checkerboard assay (Table 2). MRSA USA 300 (10^6^ CFU/mL) was treated with a combination of different concentrations of LyeTx I mnΔKL (1–32 µM) and vancomycin (1–32 µM) for 24 h at 37 °C, and the fractional inhibitory concentration (FIC) and FIC index (FICI) were determined. The combination of LyeTx I mnΔKL or LyeTx I mnΔK with vancomycin yielded a FICI of 2 (Table 2), indicating an indifferent interaction [35].

We then investigated whether the pre-treatment with LyeTx I mnΔKL alters MRSA resistance to different classes of antimicrobial agents using the MIC assay. MRSA USA 300 (10^6^ CFU/mL) was exposed to ¼ × MIC of LyeTx I mnΔKL or LyeTx I mnΔK for 1 h, followed by the determination of the MIC of oxacillin, levofloxacin, kanamycin, and erythromycin. As shown in Table 3, MRSA presented high resistance to all antibiotics tested (MIC > 128 µg/mL). Pre-exposure to LyeTx I mnΔKL reduced the MICs of oxacillin and levofloxacin to 128 µg/mL, while LyeTx I mnΔK reduced the MICs to 64 and 128 µg/mL, respectively. No resensitization effect was observed for kanamycin or erythromycin (MIC > 128 µg/mL) for both peptides.

Collectively, these results demonstrate that both LyeTx I mnΔKL and its prototype can resensitize resistant MRSA cells to β-lactams and fluoroquinolones.

### 2.5. Cytotoxicity

The cytotoxic effect of LyeTx I mnΔKL was evaluated against embryonic kidney (HEK-293) and hepatic (Hep G2) human cell lines. The cytotoxic concentration for 50% of the cells (CC_50_) and selective index (SI) values are presented in Table 4. LyeTx I mnΔKL presented CC_50_ of 5.85 and 5.51 µM for HEK-293 and Hep G2, respectively, while LyeTx I mnΔK presented CC_50_ values of 32.72 and 30.76 µM, respectively. Overall, LyeTx I mnΔKL and LyeTx I mnΔK displayed an average SI value of ~2 (2.76 and 2.60 for HEK-293 and Hep G2, respectively, for LyeTx I mnΔKL, and 2.61 and 2.46 for LyeTx I mnΔK), indicative of elevated toxicity. These results indicate that both LyeTx I mnΔKL and its prototype lack selectivity for bacterial cells. Furthermore, the insertion of a leucine increased the toxicity of the peptide, as evidenced by the CC_50_ values.

### 2.6. LyeTx I mnΔKL Induces Cell Lysis of MRSA Cells

**Figure 3:**
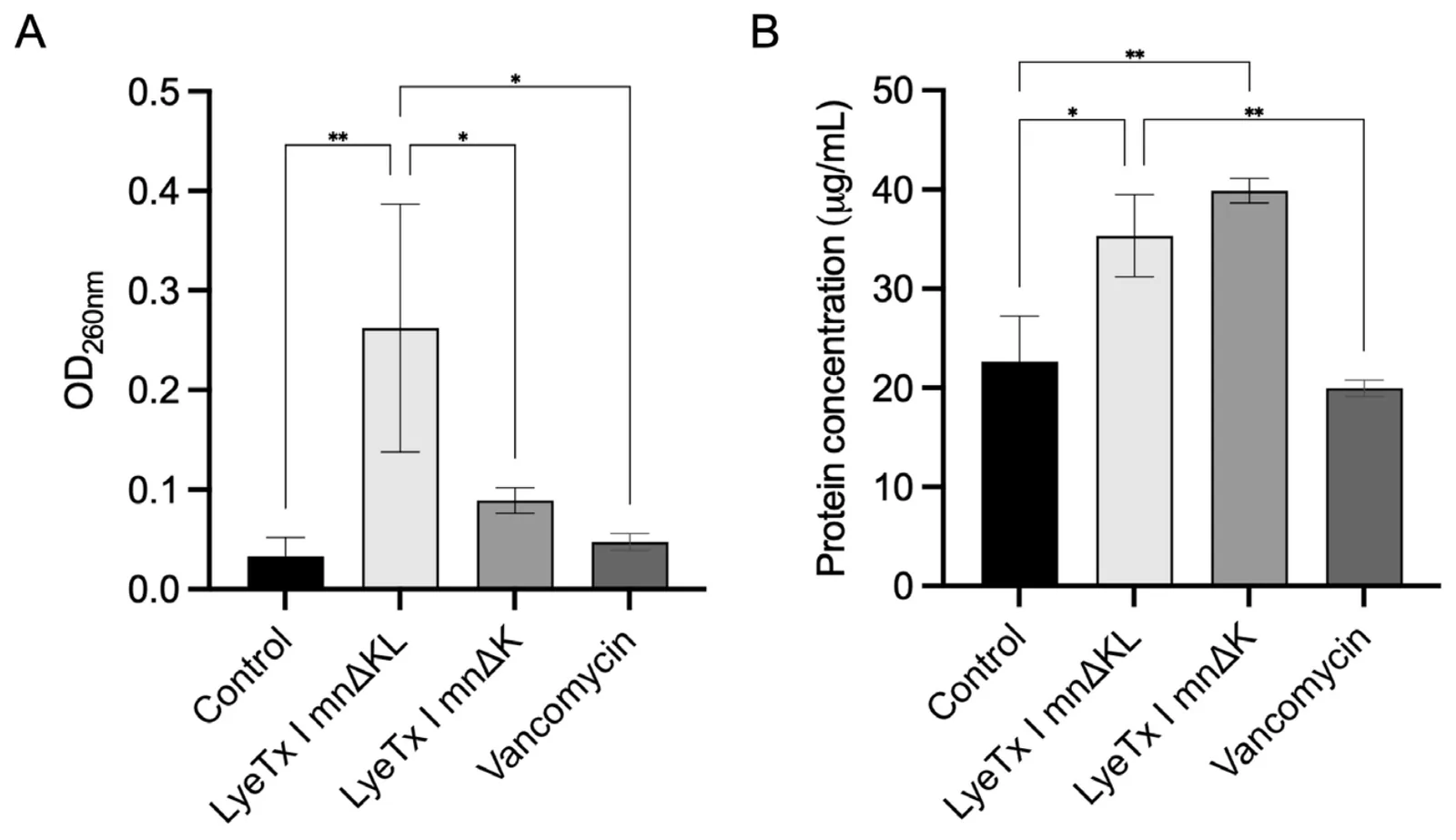
Release of intracellular material induced by LyeTx I mnΔKL treatment. (**A**) Release of nucleic acids. MRSA USA 300 (10^8^ CFU/mL) was treated with 20 μM of LyeTx I mnΔKL, LyeTx I mnΔK, or vancomycin for 24 h at 37 °C. The OD of the supernatant was measured at 260 nm. (**B**) Protein release by the Bradford assay. MRSA USA 300 (10^8^ CFU/mL) was treated with 20 μM of LyeTx I mnΔKL, LyeTx I mnΔK, or vancomycin for 24 h at 37 °C, then mixed with Coomassie Brilliant Blue for 2 min. The OD at 595 nm was measured, and protein concentrations were determined based on a standard curve created using bovine serum albumin. * *p* < 0.05; ** *p* < 0.01.

In accordance with the primary mode of action of AMPs, a previous study conducted by our group demonstrated that LyeTx I mnΔK damages the MRSA cell membrane [25]. To investigate if the addition of a leucine residue alters the mechanism of action of LyeTx I mnΔKL, we analyzed the release of intracellular material spectrophotometrically by quantifying nucleic acids and proteins in the supernatant of MRSA cells (Figure 3). To detect the release of intracellular material, MRSA USA 300 cells (10^8^ CFU/mL) were treated with a lethal concentration of LyeTx I mnΔKL (i.e., 20 µM; 10 × MIC of LyeTx I mnΔKL) for 24 h at 37 °C. Nucleic acid release was measured by spectrophotometry at 260 nm, and protein release was evaluated by the Bradford assay. Untreated cells, LyeTx I mnΔK, and vancomycin were included as controls. As shown in Figure 3A, treatment with LyeTx I mnΔKL increased the amount of nucleic acid from 0.03 in the untreated control to 0.26, representing more than an 8-fold increase. The LyeTx I mnΔK induced a smaller increase (OD_260nm_ 0.089), approximately 3-fold lower than LyeTx I mnΔKL, while vancomycin showed even lower OD values (OD_260nm_ 0.047).

To build on these results, we measured protein concentration in the supernatant of MRSA-treated cells (Figure 3B). Consistent with the nucleic acid leakage data, treatment with LyeTx I mnΔKL increased protein release from 22.6 µg/mL in the untreated control to 35.3 µg/mL. For cells treated with LyeTx I mnΔK, a high protein concentration of 39.9 µg/mL was also obtained, indicating cell lysis. In contrast, vancomycin treatment resulted in a lower protein concentration (19.9 µg/mL) (Figure 3B). Together, these findings confirm that LyeTx I mnΔKL damages the MRSA cell membrane, leading to leakage of intracellular material.

### 2.7. LyeTx I mnΔK and LyeTx I mnΔKL Interact with Bacterial Membrane-Mimetic Vesicles

**Figure 4:**
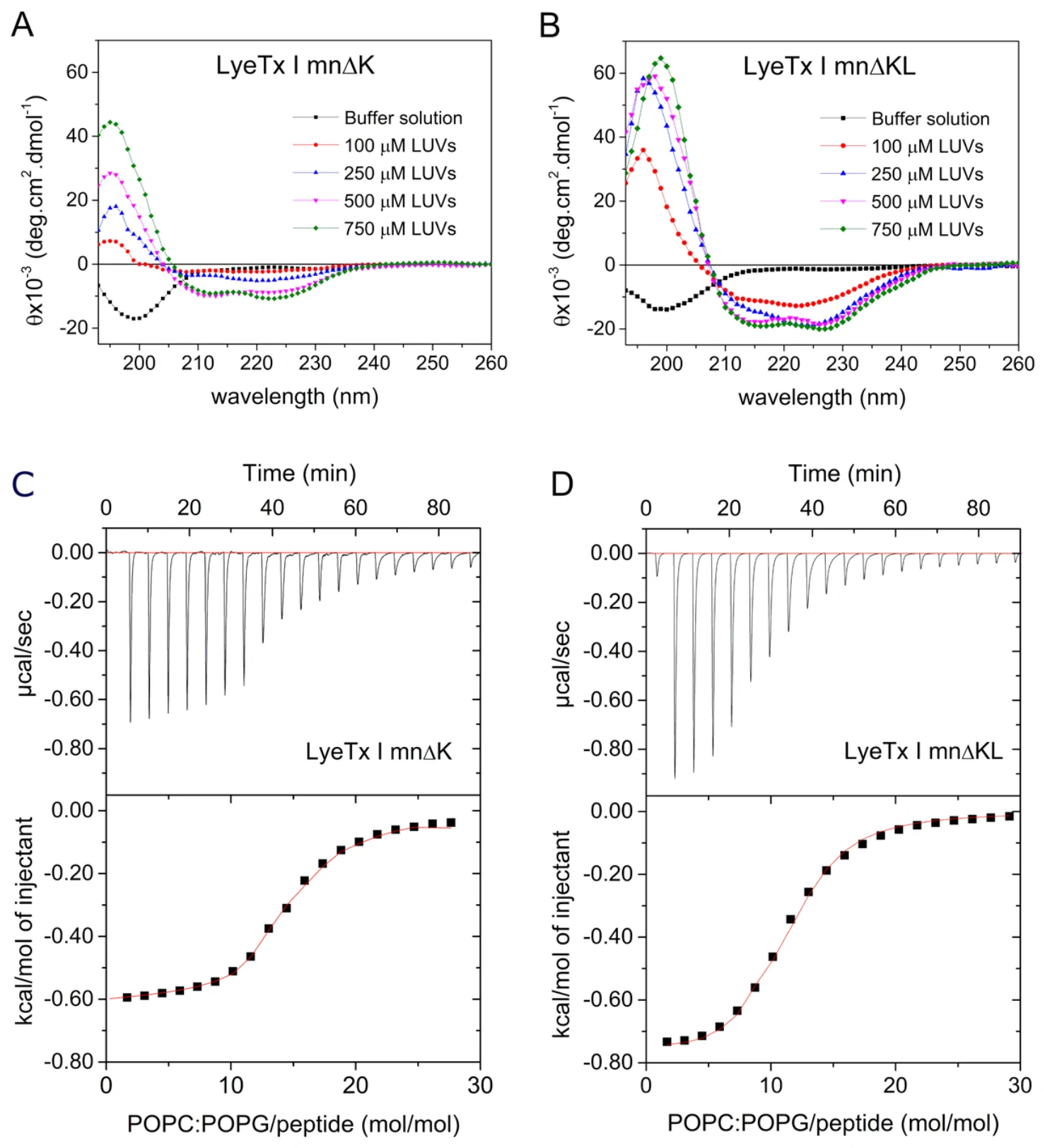
Circular dichroism (CD) spectra of (**A**) LyeTx I mnΔK (50 μM) and (**B**) LyeTx I mnΔKL (50 μM) in Tris-HCl buffer at pH 7.5 and at different POPC:POPG (3:1) mol/mol LUVs. Isothermal titration calorimetry (ITC) obtained from the titration of 20 mM POPC:POPG (3:1) in (**C**) LyeTx I mnΔK (50 μM) and (**D**) LyeTx I mnΔKL (50 μM) solutions in Tris-HCl buffer at pH 7.5 and 30 °C.

Based on the results shown in Figure 3, which indicate that LyeTx I mn∆KL damages *S. aureus* membranes, its interaction with lipid bilayers was further evaluated by CD and ITC using large unilamellar vesicles (LUVs) composed of POPC:POPG. CD spectra (Figure 4A,B) revealed that both peptides adopt predominantly disordered conformations in aqueous solution and undergo a structural transition to α-helical conformations upon interaction with POPC:POPG vesicles. This behavior is typical of amphipathic AMPs [36] as observed for LyeTx I mnΔK, which acquires a defined secondary structure upon POPG:CL membrane association [25]. The appearance of the characteristic minima at 208 and 222 nm confirmed membrane-induced folding for both analogs. However, significant differences were observed in the molar helicity (*H*) and the lipid concentration required to achieve maximal *H*. LyeTx I mnΔKL reached its maximum helical content at 250 μM POPC:POPG, whereas LyeTx I mnΔK required 500 μM lipid concentration to attain its maximum folding state. In addition, the ellipticity minima at 208 and 222 nm were more intense for LyeTx I mnΔKL (approximately −20 × 10^3^ deg·cm^2^·dmol^−1^) than for LyeTx I mnΔK (approximately −10 × 10^3^ deg·cm^2^·dmol^−1^), indicating a significantly higher α-helical content in the membrane-bound state. These results demonstrate that the insertion of a single leucine residue enhances both the membrane-induced propensity to fold and the stability of the α-helical conformation.

ITC experiments (Figure 4C,D) further demonstrated that both peptides interact favorably with POPC:POPG vesicles through spontaneous, exergonic processes driven by enthalpic and entropic contributions (Table 5). Analysis of the binding isotherms revealed similar Gibbs free energy (Δ*G*) and entropy (Δ*S*) values for both peptides, whereas notable differences were observed in the apparent association constant (*K*_app_) and enthalpy change (Δ*H*). LyeTx I mnΔKL exhibited a *K*_app_ of 3.9 × 10^4^ L·mol^−1^, approximately twofold higher than that of LyeTx I mnΔK (2.0 × 10^4^ L·mol^−1^), indicating a stronger affinity for the membrane. In addition, the enthalpic contribution associated with membrane binding was approximately three-fold greater for LyeTx I mnΔKL (Δ*H* = −730 cal·mol^−1^) than for LyeTx I mnΔK (Δ*H* = −215 cal·mol^−1^). The higher positive entropy term (TΔS) observed for both peptide–membrane interactions indicates a predominantly entropy-driven process, likely arising from the release of structured water molecules during membrane desolvation and peptide insertion into the lipid bilayer [25,37]. These findings are consistent with previous studies of LyeTx I mnΔK interacting with POPG:CL membranes [25] and further support the ability of both peptides to bind and perturb bacterial membrane-mimetic bilayers.

### 2.8. Modulation of Zeta Potential and Hydrodynamic Diameter by LyeTx I mnΔK and LyeTx I mnΔKL

**Figure 5:**
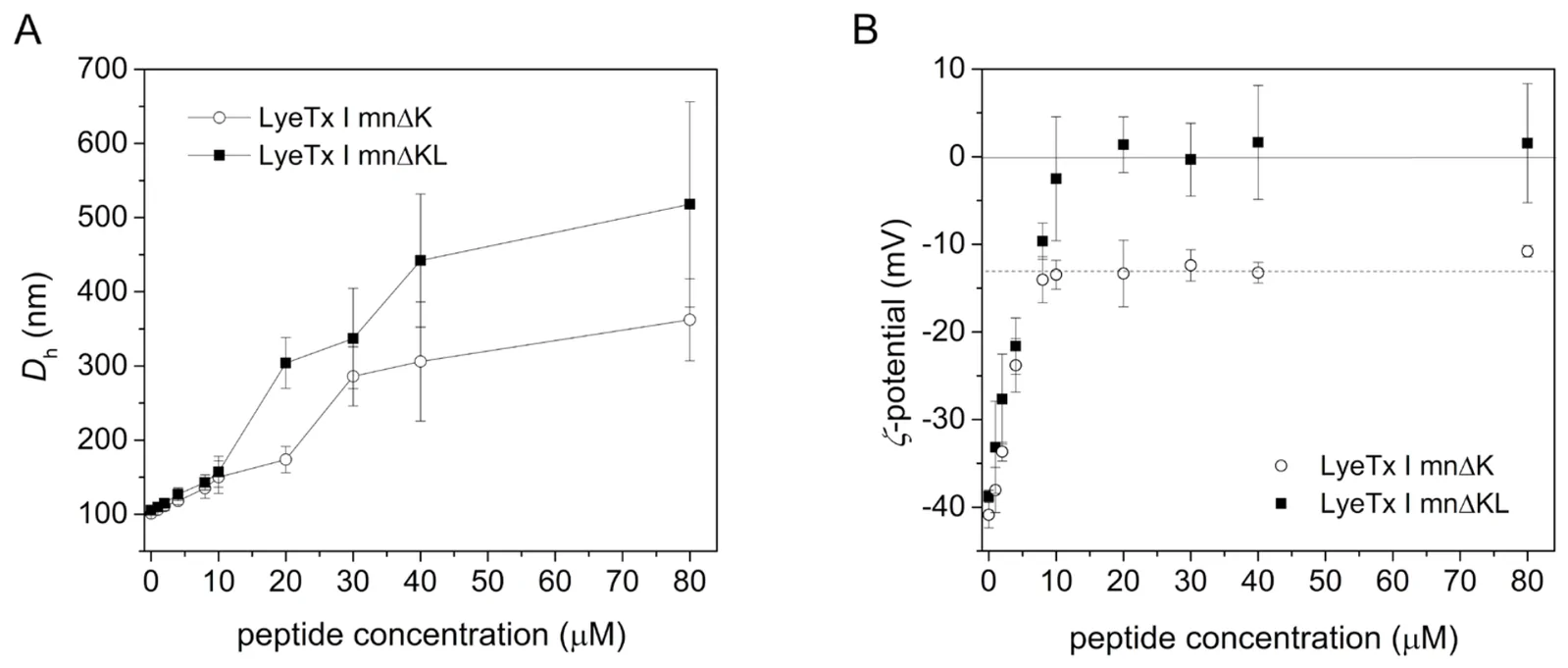
Effect of LyeTx I mn∆K and LyeTx I mn∆KL on (**A**) hydrodynamic diameter (D*_h_*) and (**B**) zeta (ζ) potential of 500 μM POPC: POPG LUVs (3:1, mol:mol).

The interaction of LyeTx I mn∆KL and LyeTx I mn∆K with POPC vesicles was further investigated by monitoring changes in hydrodynamic diameter (D*_h_*) and zeta potential (ζ) (Figure 5). No statistically significant differences were observed in the D*_h_* of vesicles (Figure 5A) treated with the peptides, indicating that both analogs induce comparable effects on vesicle size and overall aggregation behavior. Both peptides showed an increase of approximately 50 nm up to 10 µM, reflecting the surface-associated peptide–membrane interaction. Interestingly, at peptide concentrations above 10 µM, the D*_h_* exceeded 200 nm with increased polydispersity (0.9), suggesting vesicle aggregation or fusion events [38]. These results are in line with previous observations in POPG:CL:LysylPOPG systems [25] and reflect peptide-induced membrane perturbation of the anionic vesicles.

In contrast, differences were detected in the modulation of surface charge. Pure POPC:POPG vesicles exhibited an initial ζ-potential of −39.0 ± 1.5 mV (Figure 5B), consistent with their anionic composition [39]. Progressive addition of LyeTx I mn∆KL shifted the ζ-potential toward less negative values, reaching neutral charge (0.5 ± 3.0 mV) at the highest peptide concentration, indicating effective surface charge neutralization via electrostatic adsorption of the peptide. Conversely, LyeTx I mnΔK induced only a partial neutralization of the vesicle surface, increasing the ζ-potential to approximately −10 mV (Figure 5B). These results demonstrate that the additional leucine residue substantially enhances the peptide’s ability to interact with and protect the negatively charged membrane surface.

### 2.9. LyeTx I mnΔKL Does Not Induce Oxidative Stress in MRSA Cells

The ability of LyeTx I mn∆KL to induce oxidative stress in MRSA cells was indirectly assessed by MIC determination in the presence of ascorbic acid. An increase in the MIC by at least two dilutions is considered indicative of oxidative stress damage. LyeTx I mnΔKL did not induce significant oxidative stress on MRSA cells (Table 6), with MIC values post-ascorbic acid supplementation of 4 μM compared to the control (2 μM). On the other hand, LyeTx I mnΔK induced pronounced oxidative stress in MRSA cells, resulting in an 8-fold increase in the MIC upon supplementation with ascorbic acid. These results suggest that oxidative stress induction is possibly not a mode of action of LyeTx I mnΔKL.

### 2.10. LyeTx I mnΔKL Reduces Bacterial Burden in a Murine Wound/Abscess Infection Model

**Figure 6:**
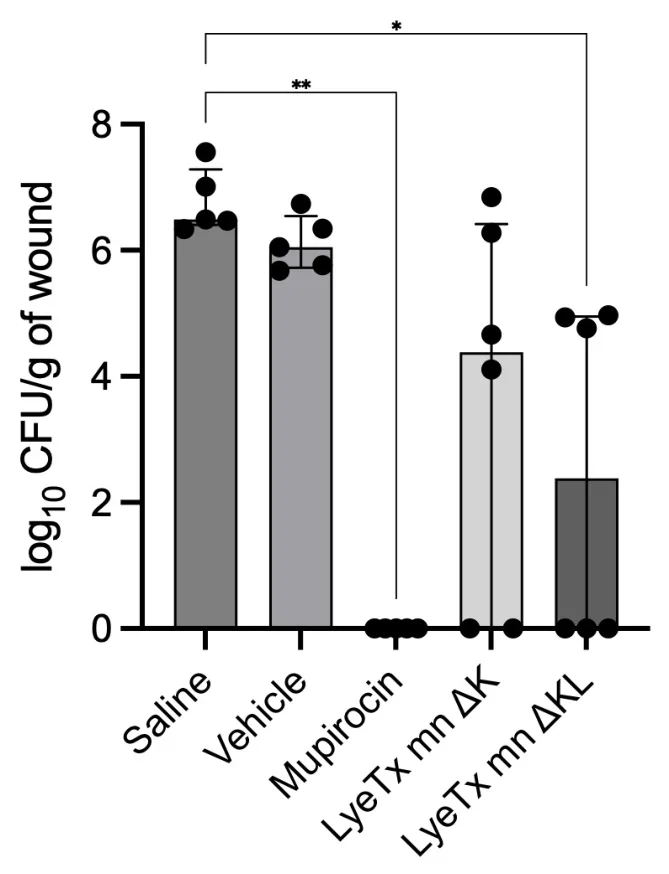
In vivo effect of LyeTx I mnΔKL in a mouse wound/abscess model induced by MRSA. Mice were infected with MRSA at 10^8^ CFU/mL in the dorsal area. Forty-eight h after inoculation, the animals were topically treated with 0.5% of LyeTx I mnΔKL, LyeTx I mnΔK, or mupirocin daily for 3 days. Saline solution and gel formulation were used as controls. * *p* < 0.05; ** *p* < 0.01.

Considering that MRSA is one of the leading causes of skin infections [40] and that LyeTx I mnΔKL showed enhanced anti-MRSA activity (Table 1), we evaluated its topical efficacy using a murine wound/abscess infection model. Five-week-old mice were infected with MRSA USA 300 (10^8^ CFU/mL), and treatment with 0.5% (*w*/*w*) of LyeTx I mnΔKL, LyeTx I mnΔK, and mupirocin was initiated 48 h post-infection and continued daily for three days. Mupirocin was used as the positive control, and its MIC and MBC against the MRSA strains used in this study are shown in Supplementary Table S1. 0.5% (*w*/*w*) was used, as a previous study by our group showed that LyeTx I mnΔK exhibited the most pronounced in vivo effect at this concentration [25]. A significant reduction in bacterial load was observed in the peptide-treated groups, with log_10_ CFU/g of wound of 2.4 for LyeTx I mnΔKL and 4.4 for LyeTx I mnΔK, compared to 6.5 and 6.04 in the saline and formulation gel groups, respectively. Furthermore, LyeTx I mnΔKL induced complete bacterial elimination in 50% of the animals (3/6), compared with 33% (2/6) for the prototype (i.e., LyeTx I mnΔK) (Figure 6). Complete elimination of MRSA cells was observed with mupirocin (Figure 6), an effective agent for treating skin infections, confirming its potent antimicrobial activity and validating our experimental conditions.

## 3. Discussion

Although significant progress has been made over the past decades in characterizing the antimicrobial effect of AMPs and their analogs, to date, only a few have been approved for clinical use (i.e., gramicidin, colistin, polymyxin B, daptomycin, vancomycin, oritavancin, dalbavancin, and telavancin) [41]. This is attributed to several challenges associated with the use of these molecules, including high synthesis costs, low stability under physiological conditions, and potential toxicity to human cells [42]. In this study, we designed a novel AMP molecule, LyeTx I mnΔKL, derived from the natural peptide LyeTx I [26] and the synthetic modified AMP LyeTx I mnΔK [28]. We showed that LyeTx I mnΔKL presented increased in vitro and in vivo antimicrobial and killing effects against MRSA clinical isolates compared to its prototype (i.e., LyeTx I mnΔK). Furthermore, similar to LyeTx I mnΔK, we show that LyeTx I mnΔKL acts on the membrane of *S. aureus*, possibly as its primary mode of action. However, the peptide presented high toxicity. Importantly, we demonstrate that LyeTx I mnΔKL affects biofilm formation and mature MRSA biofilms.

The natural peptide LyeTx I, derived from a *L. erythrognatha* toxin, was first isolated and its antimicrobial activity described by our group in 2010 [26]. It exhibited antimicrobial activity against *E. coli* (MIC 7.81 μM), *S. aureus* (MIC 3.79 μM), *P. kudriavzevii* (MIC 26.3 μM), and *C. neoformans* (MIC 13.2 μM), with a more pronounced effect on Gram-positive bacteria. However, its hemolytic activity at high concentrations and relatively long chain (25 amino acid residues) may limit its applicability as an antimicrobial agent. Then, in a follow-up study, three shortened LyeTx I analogs were synthesized: LyeTx I mn with 15 amino acid residues, LyeTx I mnΔK with a lysine inserted at position 5, and its acetylated variation LyeTx I mnΔKAc. Although LyeTx I showed an increased antimicrobial effect against Gram-negative, Gram-positive, and fungal species compared to its analogs, the synthetic derivatives showed reduced hemolytic activity. Moreover, LyeTx I and all three analogs were shown to adopt an α-helical conformation and exhibit membranolytic effects. Among the analogs studied, LyeTx I mnΔK presented more promising effects with higher antimicrobial activity and lower cytotoxicity [28]. Consistently, follow-up studies demonstrated the activity of LyeTx I mnΔK against carbapenem-resistant *A. baumannii* (CRAB) strains (MIC_50_ of 4 μM) by disrupting bacterial cell membranes [23]. Importantly, LyeTx I mnΔK showed lower cytotoxicity and hemolytic activities compared to LyeTx I [23]. It also exhibited promising antifungal activity against *Candida* species [29] and efficacy against MRSA cells [25]. The reduction in antimicrobial potency of LyeTx I mnΔK compared to LyeTx I could be attributed to decreases in net positive charge and hydrophobicity, as well as to the overall reduction in peptide size. However, LyeTx I mnΔK displayed low stability in fetal bovine serum and human plasma [23], which may impact its potential applicability. In addition, PEGylation may represent a valuable strategy to improve the in vivo stability of these peptides while reducing their toxicity, as previously demonstrated for LyeTx I-b, another derivative of LyeTx I, which showed efficacy against multidrug-resistant *A. baumannii* pneumonia in mice [43].

In accordance with our studies, it has been previously shown that adding leucine residues to peptides can increase the helicity potential [31] and hydrophobicity, promoting membrane permeabilization [30] and enhancing biological activity [31]. Indeed, leucine zipper sequences have been reported as promising modulators of toxicity and antimicrobial activity [44]. In this context, the rapid killing observed in the kill-curve assay for LyeTx I mnΔKL compared to LyeTx I mnΔK and vancomycin, which is known to have limited bactericidal activity [45], could be attributed, at least in part, to the leucine addition to the peptide structure. In fact, the presence of carboxyamidated leucine at the *C*-terminal position of cationic short-chain peptides obtained from the toxins of insects of the order Hymenoptera [46,47,48], as well as most proline-rich peptides from insects in general [49], appears to be a mandatory structural requirement for the antimicrobial activity of these natural products.

Biofilms are recognized as the predominant form of bacteria in nature and represent a chemical and physical barrier against environmental stressors [32,50,51]. In addition to the presence of an extracellular polymeric substance matrix, the increased resistance of biofilms to antimicrobial agents and host immune factors is attributed to multiple mechanisms, including the presence of persister cells, low-metabolic or non-replicating cells, and gradients of oxygen and nutrients [33]. Therefore, the development of antimicrobial agents that target not only mature biofilms but also the initial stages of biofilm formation represents a promising strategy to enhance infection control. Here, we show that the modified peptide LyeTx I mnΔKL had a more pronounced effect on biofilm formation and 24-h MRSA biofilms than LyeTx I mnΔK. In addition to biomass quantification, analysis of biofilm cell viability revealed that LyeTx I mnΔKL reduced the viability of 24-h biofilms at the highest concentrations tested (i.e., 4 and 10 × MIC), compared to LyeTx I mnΔK, which was effective only at 10 × MIC. In accordance with our results, LyeTx I was previously shown to reduce the biomass of 24- and 48-h *A. baumannii* [23] and *C. albicans* [29] biofilms. Among the mechanisms involved in the antibiofilm effect of AMPs are the downregulation of adhesion genes, cell-surface modification (e.g., hydrophobicity and charge), and bacterial cell killing [52]. Given that LyeTx I mnΔKL affected only the viability of 24-h biofilms, we hypothesize that this modified AMP may act on non-growing cells. Indeed, the limited efficacy of traditional antibiotics in eradicating biofilms is, at least in part, due to their poor activity against slow-growing or stationary-phase cells [53,54]. The enhanced activity of LyeTx I mnΔKL could also involve interactions with components of the biofilm matrix. For instance, cationic antibiotics, such as colistin, polymyxin B, and tobramycin, interact electrostatically with the biofilm matrix of *P. aeruginosa*, quenching its components and enhancing the antimicrobial activity of the antibiotics [55,56].

The primary mode of action of AMPs involves the interaction with bacterial membranes, leading to membrane disruption and cell lysis [57]. In accordance with previous studies demonstrating the membranolytic activity of LyeTx I and LyeTx I mnΔK [23,25,28], the novel analog LyeTx I mnΔKL presented a strong affinity for anionic lipid membranes, a key feature of AMPs [28]. However, biophysical characterization revealed important differences between LyeTx I mnΔK and LyeTx I mnΔK L that help explain the enhanced antimicrobial activity of LyeTx I mnΔKL. CD spectroscopy showed that both peptides underwent a transition from a predominantly disordered conformation in aqueous solution to an α-helical structure upon interaction with POPC vesicles. Such membrane-induced folding is a common feature of amphipathic AMPs and is generally associated with membrane insertion and disruption [58]. Notably, LyeTx I mnΔKL showed a markedly higher α-helical content in the membrane-bound state. These findings suggest that the additional C-terminal leucine promotes a more efficient membrane-induced folding process and stabilizes the amphipathic α-helical conformation, thereby favoring peptide partitioning into the lipid bilayer.

Thermodynamic data obtained by ITC further support this interpretation. The dominant positive entropy contribution observed for both peptides is consistent with membrane desolvation resulting from peptide–membrane association [59]. Although both peptides exhibited favorable, predominantly entropy-driven interactions with POPC:POPG vesicles, LyeTx I mnΔKL displayed an apparent association constant approximately twofold higher than that of LyeTx I mnΔK. Moreover, the enthalpic contribution associated with membrane binding was nearly three-fold greater for LyeTx I mnΔKL.

Further evidence of enhanced membrane interaction was provided by *D_h_* and ζ-potential measurements. The increase in ζ-potential and *D_h_* indicates not only electrostatic interactions of both peptides but also deeper insertion and membrane remodeling. The observed enlargement of vesicle size suggests fusion or aggregation, driven by peptide–membrane interactions [60]. While LyeTx I mnΔKL and LyeTx I mnΔK induced changes in the surface charge of POPC vesicles, LyeTx I mnΔKL nearly completely neutralized the negative membrane surface, whereas LyeTx I mnΔK produced only partial neutralization, reaching approximately −10 mV at the highest concentration tested. This greater charge neutralization is consistent with higher membrane affinity observed by ITC and the increased membrane-induced helicity detected by CD.

Collectively, the CD, ITC, and ζ-potential data establish the impact of structural modification on peptide–membrane interactions and the improved biological activity of LyeTx I mnΔKL. These results corroborate the findings of Vieira et al. (2024) [25] and reinforce that LyeTx I mn∆KL exerts its antimicrobial activity by targeting the bacterial membrane, leading to physical destabilization. The addition of a single leucine residue enhances membrane affinity and increases membrane charge neutralization, ultimately leading to more efficient membrane perturbation. These properties likely contribute to the lower MIC values, faster bactericidal kinetics, and improved antibiofilm activity observed for LyeTx I mnΔKL relative to its precursor peptide.

The membranolytic activity of LyeTx I mnΔKL could also explain the rapid killing observed in the kill-curve assays and its efficacy against biofilms [52]. Furthermore, the membrane-targeting mechanism of AMPs limits the propensity of resistance induction, an important advantage over conventional antimicrobial agents [42]. Supporting this, previous studies showed that exposure to sublethal concentrations of LyeTx I mnΔK did not induce resistance in *A. baumannii* [23] or *C. albicans* [29] over 14 and 21 days, respectively. In the study by Lima et al. (2021), colistin, the positive control used, induced a 4-fold increase in the MIC of *A. baumannii* after 14 days of exposure [23].

Additionally, the antimicrobial effect of several antibiotics, such as ampicillin, kanamycin, norfloxacin, and ciprofloxacin, is also mediated by the generation of oxidative stress [61,62,63] by varied mechanisms, including induction of the Fenton reaction [62]. The accumulation of these toxic reactive oxygen species (ROS) damages several cellular molecules, leading to cell death [64]. To expand on the other possible modes of action of LyeTx I mnΔKL, we tested if it induces oxidative stress in MRSA cells by the indirect ascorbic acid method. We showed that, although no effect of LyeTx I mnΔKL on oxidative stress was observed, LyeTx I mnΔK induced oxidative stress in MRSA cells. These results show that the antimicrobial mechanism of action of the prototype was altered after the addition of leucine to its *C*-terminal region. Thus, future studies will focus on an in-depth characterization of alternative modes of action of LyeTx I mnΔKL.

While the membranolytic effect of AMPs confers several advantages, as discussed above, it also contributes to a significant challenge in using AMPs in the clinic—toxicity. Although the peptide structure modification increased the antibacterial effect of LyeTx I mnΔKL, it presented increased toxicity compared to the prototype [23,25,28].

A promising alternative widely explored in antimicrobial therapy is the combination of antimicrobial agents to increase treatment efficacy while reducing their required dose. For instance, membrane-targeting agents are known to disrupt the cell membrane, facilitating the entry of a second antimicrobial into the cell, where it can act on intracellular targets [65]. In our analysis, the combination of LyeTx I mnΔKL or of the LyeTx I mnΔK with vancomycin did not enhance the antimicrobial activity of either agent. However, the resensitizing effect of LyeTx I mnΔKL on MRSA cells to oxacillin and levofloxacin could make this AMP still an option for combination therapy or as an adjuvant, as resensitization is currently under exploration [6].

The toxicity of LyeTx I mnΔKL against kidney and hepatic cells is not a limiting factor for its potential use as an antimicrobial agent. Therefore, given that MRSA is an important pathogen associated with skin and wound infections and that the treatment options for this condition are limited [11,66], the topical use of LyeTx I mnΔKL as a gel was evaluated. The peptide presented a promising effect in reducing MRSA load in wound/abscess infections compared to the prototype. In accordance, LyeTx I mnΔK was previously shown to produce a similar anti-MRSA effect in murine wounds/abscesses at 0.25, 0.5, and 1% in a dose-independent manner [25]. Supporting the promising use of LyeTx I derivatives, a mouse model of arthritis induced by *S. aureus* showed that LyeTx I mnΔK reduced bacterial load and pain and inflammation in mice [28], and the use of this AMP to treat CRAB-induced pneumoniae significantly reduced bacterial load in the lungs [23].

Taken together, our results show, for the first time, that LyeTx I mnΔKL is a promising compound for the development of new antibacterial agents against MDR infections. We show that modifying an AMP molecule by adding a leucine residue drastically increased the peptide’s antimicrobial activity in vitro and in vivo. Despite these promising results, this study has limitations. Although cytotoxicity was evaluated in HEK-293 and HepG2 cells, toxicity was not assessed in skin-relevant cell lines, such as keratinocytes or primary dermal fibroblasts, despite the use of a cutaneous infection model. In addition, membrane damage was evaluated indirectly through the extracellular release of nucleic acids and proteins, whereas a direct membrane-permeabilization assay, such as propidium iodide uptake, was not performed.

## 4. Materials and Methods

### 4.1. Bacterial Strains, Cell Lineages, and Growth Conditions

MRSA USA 300 and clinical isolates were used in this study. MRSA USA 300 was kindly provided by the Reference Laboratory in Microbiology of the Oswaldo Cruz Foundation (FIOCRUZ-RJ, Rio de Janeiro, Brazil), and clinical isolates (identified as 11, 29, 49, 130, 472, 659, and 685) were obtained from wound infections of patients admitted to the Santa Casa Hospital of Belo Horizonte (Belo Horizonte, MG, Brazil). Clinical isolates were identified by standard morphological and biochemical methods, and the MRSA phenotype was confirmed by disk-diffusion testing using a 30 µg cefoxitin disk, according to BrCAST guidelines. Isolates with an inhibition-zone diameter of <25 mm were classified as MRSA [67]. MRSA strains were maintained on mannitol salt agar (MSA; Kasvi, Brazil) and cultured on Mueller–Hinton agar (MHA; Kasvi, Brazil) for 24 h at 37 °C before the experiments.

For cytotoxicity assays, human embryonic kidney epithelial cells (HEK-293) and human hepatocellular carcinoma epithelial cells (HepG2) were used. Cells were cultured in Dulbecco’s Modified Eagle’s Medium (DMEM) supplemented with 5% fetal bovine serum (FBS), L-glutamine (50 mg/mL), and 0.3% penicillin-streptomycin-amphotericin B solution (10,000 U/mL + 10 mg/mL + 2 mg/mL, respectively) at 37 °C in a 5% CO_2_ atmosphere.

Stock solutions of vancomycin (Sigma-Aldrich, São Paulo, Brazil) and peptides were prepared in sterile distilled water at a concentration of 1.28 mM. Stock solutions were divided into single-use aliquots and stored at −20 °C until use.

### 4.2. Peptide Synthesis and Purification

LyeTx I mn∆KL and LyeTx I mn∆K were manually synthesized using the Fmoc-based solid-phase peptide synthesis strategy [68] on Rink-amide resin (substitution degree: 0.60 mmol·g^−1^). Successive coupling steps were carried out in a solution containing 1.5 mL of N,N-dimethylformamide (DMF) and activating agents, such as 1,3-diisopropylcarbodiimide (DIC) and 1-hydroxybenzotriazole (HOBt), under constant stirring (240 rpm) for 2 h. Deprotection reactions were performed using a 25% (*v*/*v*) 4-methylpiperidine/DMF solution in two 15-min steps. The deprotection and coupling processes were monitored using a qualitative Kaiser test [69]. Peptide cleavage from the resin was achieved with a TFA/triisopropylsilane/water (95:2.5:2.5, *v*/*v*/*v*) solution under stirring for 1 h and 30 min. Subsequently, the peptide was precipitated with diisopropyl ether, dissolved in water, and lyophilized.

The purity of LyeTx I mn∆K and LyeTx I mn∆KL was evaluated using high-performance liquid chromatography and mass spectrometry (Supplementary Figure S1). (HPLC). A mobile phase gradient consisting of acetonitrile/TFA (0.08%, *v*/*v*) and water/TFA (0.1%, *v*/*v*) was used. The analysis was performed on a Pro Star^®^ 315 chromatograph (Varian^®^, Inc., Walnut Creek, CA, USA), equipped with a 100 μL loop and a Jupiter 4 Mm Proteo 90 Å, LC column 250 × 10 mm (Phenomenex, Inc., Torrance, CA, USA). A 500 μL injection of the crude sample at 5 mg/mL was carried out at a flow rate of 3 mL/min. Elution was achieved using a linear gradient of acetonitrile (35 to 47.5%) over 25 min.

### 4.3. Antibacterial Assay

The MIC was determined by the broth microdilution method in MHB supplemented with 0.002% Tween-80^TM^ (Kasvi, Pinhais, Brazil), according to Document M07 of the Clinical and Laboratory Standards Institute [70]. Briefly, isolated colonies of MRSA grown on MHA were suspended in 0.9% saline solution and adjusted to a 0.5 McFarland standard (~10^8^ CFU/mL). The bacterial suspension was then diluted in MHB to obtain a final inoculum of 10^6^ CFU/mL. Standard twofold serial dilutions of the antimicrobial agents (0.125–64 μM) were prepared in MHB, and the plates were incubated at 37 °C for 24 h. Bacterial suspensions incubated in MHB without antimicrobial agents served as the growth control, while MHB without bacteria was used as the sterility control. Vancomycin was included as the reference antimicrobial. The plates were incubated at 37 °C for 24 h, and the MIC was defined as the lowest concentration of an antimicrobial agent that completely inhibited visible bacterial growth.

The MBC was determined by transferring 50 μL from MIC wells in which no visible growth was detected onto MHA plates, followed by incubation at 37 °C for 24 h. The MBC was defined as the lowest concentration that reduced bacterial growth by 99% compared to the untreated control.

### 4.4. Time-Kill Kinetics

The effect of LyeTx I mnΔKL on MRSA cells over time was assessed by CFU determination. Isolated colonies of MRSA USA 300 were resuspended in 0.9% saline, adjusted to the 0.5 McFarland standard (~10^8^ CFU/mL), and diluted 1:200 in MHB (~10^6^ CFU/mL). The cells were treated with 20 μM of the antimicrobial agents, corresponding to 10 × MIC of LyeTx I mnΔKL, for 3 h at 37 °C. After 30, 60, 90, 120, 150, and 180 min of incubation, aliquots of 100 μL were collected, serially diluted, and plated out on MSA. The plates were incubated for 24 h at 37 °C and the CFU/mL determined. LyeTx I mnΔK-, untreated-, and vancomycin-treated cells were used as controls.

### 4.5. Effect of LyeTx I mnΔKL on Biofilm Formation and Mature Biofilms

The effect of LyeTx I mnΔK and LyeTx I mnΔKL on MRSA biofilm formation and mature biofilms was evaluated by crystal violet staining [71] and cell viability assays [72]. Isolated colonies of MRSA USA 300 were resuspended in 0.9% saline and adjusted to the 0.5 McFarland standard (~10^8^ CFU/mL), followed by a 1:200 dilution in MHB supplemented with 1% glucose (10^6^ CFU/mL). For the biofilm formation inhibition assay, MRSA was inoculated into 96-well microtiter plates and incubated with sub-lethal concentrations of the antimicrobial agents (1/8 ×, ¼ ×, and ½ × MIC) for 24 h at 37 °C under static conditions to allow biofilm establishment. For the mature biofilm inhibition assay, MRSA was transferred to 96-well microtiter plates and incubated for 24 h at 37 °C under static conditions to allow biofilm formation. Wells were then treated with the antimicrobial agents at 2 ×, 4 ×, and 10 × MIC for 24 h at 37 °C. The medium was removed, and biofilm cells were washed and fixed with methanol for 5 min at 37 °C. Biofilms were then washed and stained with 0.1% crystal violet solution for 30 min at room temperature. Glacial acetic acid at 33% was added, and the OD_595nm_ was measured using a microplate reader (Bio-Tek Instruments, Winooski, VT, USA). Untreated and vancomycin-treated cells were used as negative and positive controls, respectively.

Cell viability of biofilms was assessed by the MTT assay, as described above.

### 4.6. Cytotoxicity Assay

The cytotoxic effect of LyeTx I mnΔK and LyeTx I mnΔKL was evaluated using HEK-293 and HepG2. Cells were cultivated in DMEM supplemented with 5% FBS and antimicrobial agents at 37 °C in a 5% CO_2_ atmosphere to a confluence of ~80%. Approximately 3 × 10^4^ cells were seeded into 96-well microtiter plates and exposed to LyeTx I mnΔKL, diluted in DMEM at concentrations ranging from 0.5 to 100 μM, for 24 h at 37 °C. Cell viability was assessed by the MTT assay [73]. The cytotoxic concentration for 50% of the cells (CC_50_) and the selective index (SI; ${CC}_{50}/MIC$) were determined.

### 4.7. Combination Effect by the Checkerboard Assay

The synergistic effect of the combination of LyeTx I mnΔKL with vancomycin was evaluated by the checkerboard assay, as previously described [35,74]. LyeTx I mnΔK was used as a control. Serial dilutions of the peptides (1–32 μM) were prepared in MHB, mixed at a 1:1 ratio, and added to MRSA USA 300 (10^6^ CFU/mL), followed by incubation at 37 °C for 24 h. The FICI was calculated as the sum of the FIC of the peptide and the FIC of vancomycin, in which each FIC is defined as the MIC of the compound in combination divided by the MIC of the compound alone. The interaction was classified as synergistic if FICI ≤ 0.5, additive if 0.5 < FICI ≤ 1.0, indifferent if 1.0 < FICI ≤ 4.0, and antagonistic if FICI > 4.0 [35].

### 4.8. Effect of Pre-Exposure on Antimicrobial Susceptibility

The effect of pre-exposure of MRSA USA 300 to LyeTx I mnΔK or LyeTx I mnΔKL on antimicrobial resistance was evaluated by MIC determination. Briefly, MRSA USA 300 (10^6^ CFU/mL) was incubated with a sub-lethal concentration of ¼ × MIC of the peptides for 1 h at 37 °C. Cells were collected by centrifugation and resuspended in MHB. The MIC of oxacillin, levofloxacin, kanamycin, and erythromycin was determined as described above.

### 4.9. Evaluation of Oxidative Stress Induction

To evaluate whether LyeTx I mnΔK and LyeTx I mnΔKL induce oxidative stress in MRSA cells, ascorbic acid, a strong antioxidant [75], was used. For this, the MIC was determined using MHB supplemented with 100 μg/mL ascorbic acid, as described above.

### 4.10. Release of Intracellular Material

The release of intracellular material was evaluated spectrophotometrically by measuring the leakage of nucleic acids and proteins, as previously described [23]. For the release of nucleic acids, MRSA USA 300 (~10^8^ CFU/mL) was treated with 20 μM of LyeTx I mnΔK, LyeTx I mnΔKL, and vancomycin for 24 h at 37 °C. This lethal concentration represents 10 × MIC of LyeTx I mnΔKL. Cells were centrifuged at 3000× *g* for 25 min at 4 °C, and the absorbance of the supernatant was recorded at 260 nm using a spectrophotometer (Bio-Tek Instruments, Winooski, USA).

Protein leakage was evaluated by the Bradford method [76]. Briefly, 150 μL of MRSA USA 300 cells at 10^8^ CFU/mL, treated with 20 μM of the antimicrobial agents for 24 h at 37 °C was mixed with Coomassie Brilliant Blue G-250 for 2 min. The optical density (OD) at 595 nm (OD_595nm_) was measured using a spectrophotometer (Bio-Tek Instruments; Winooski, USA), and protein concentrations were determined using a bovine serum albumin standard curve. Untreated and vancomycin-treated cells were used as negative and positive controls, respectively.

### 4.11. Vesicle Preparation

Large unilamellar vesicles (LUVs) were obtained using the dehydration-rehydration technique, as described by Kirby and Gregoriadis (1984) [77]. A phospholipid mixture of POPC:POPG (1-palmitoyl-2-oleoyl-sn-glycero-3-phosphocholine: 1-palmitoyl-2-oleoyl-sn-glycero-3-phosphoglycerol) at a 3:1 (mol:mol) ratio was used to mimic bacterial membranes. Lipids were dissolved in chloroform, and the solvent was evaporated under a nitrogen gas stream to form a lipid film. This film was then rehydrated with Tris-HCl buffer (10 mM) containing NaCl (20 mM), pH 8.5. The resulting multilamellar vesicles underwent five cycles of freezing and thawing at 40 °C, followed by sonication, to form unilamellar vesicles. Finally, extrusion through polycarbonate membranes (100 nm) was performed to ensure uniform vesicle size, suitable for studies involving antimicrobial peptides.

### 4.12. Circular Dichroism (CD)

Conformational studies of both LyeTx I mn∆K and LyeTx I mnΔKL were investigated by CD in the presence of POPC:POPG (3:1, mol:mol) vesicles, in 20 mM Tris-HCl buffer (pH 7.5). Samples contained 50 μM peptide and increasing concentrations of phospholipids. Spectra were recorded at 30 °C using a JASCO^®^ J-810 spectropolarimeter (JASCO Corporation, Hachioji, Tokyo, Japan) equipped with a Jasco^®^ PFD-425S Peltier temperature control system (JASCO Corporation, Hachioji, Tokyo, Japan), using a wavelength range of 190−260 nm. Similar experiments were conducted with the peptide in 20 mM Tris-HCl (pH 7.5). For all experiments, blank solutions were prepared, and the resulting spectra were subtracted from each sample measurement.

### 4.13. Isothermal Titration Calorimetry (ITC)

ITC was employed to investigate the interaction of LyeTx I mn∆K and LyeTx I mnΔKL with membrane-mimetic environments. The titrations consisted of 30 successive 2-s injections of 5 μL of 50 μM peptide solutions into 20 mM POPC:POPG (3:1, mol:mol) LUVs in 10 mM aqueous Tris-HCl buffer (pH 7.5) at 30 °C. The interval between the injections was 250 s. All solutions were previously degassed using an ultrasonic bath and vacuum (140 mbar, 5 min) to remove air bubbles. ITC experiments were performed on a Malvern^®^ VP-ITC microcalorimeter (Malvern Panalytical Ltd., Malvern, United Kingdom), and the isotherms were processed and analyzed using Microcal Origin^®^ 6.0 software for ITC (Wellesley Hills, MA, USA).

### 4.14. Dynamic Light Scattering and Zeta Potential (ζ)

The effects of LyeTx I mnΔKL and LyeTx I mnΔK on the hydrodynamic diameter (D*_h_*) and zeta potential (ζ-potential) of POPC (3:1, mol) LUVs were evaluated at 25 °C using a Zetasizer Nano ZS Malvern model BI-900 (Malvern Panalytical Ltd., Malvern, United Kingdom). Measurements were performed in 700 µL polyethylene cuvettes (model DTS1060) using a 4 mW He-Ne laser (λ = 633 nm) for light scattering detection. 500 μL of LUVs (500 μM) were incubated, and the peptide was added at 15-min intervals after each addition to allow system stabilization prior to measurement. All experiments were performed in triplicate.

### 4.15. Peptide Formulation

The peptide formulation used for the in vivo assay consisted of a gel prepared according to the 6th edition of the Brazilian Pharmacopoeia [78], containing components for gel stabilization and preservation (Table 7). Methylparaben (Nipagin^®^; Fragon, Curitiba, SC, Brazil) was dissolved in water (70 °C), and hydroxyethylcellulose (Natrosol^®^; Fragon, Curitiba, SC, Brazil) was added under constant stirring to form the gel. The preparation process alternated between stirring and resting periods, resulting in a colorless gel with a pH of 6. Sodium metabisulfite was included as an antioxidant.

### 4.16. Murine MRSA Wound/Abscess Infection Model

Five-week-old female Balb/c mice (Biotério Central da UFMG, Belo Horizonte, MG, Brazil) were used in this study. All animal procedures were approved by the Laboratory Animal Research Ethics Committee of the Faculdade de Saúde Santa Casa de Belo Horizonte (CEUA-Santa Casa: 001-2023). A murine wound/abscess infection model was used, as previously described [79,80]. Initially, mice were anesthetized with ketamine (60 mg/kg) and xylazine (8 mg/kg) via intraperitoneal injection, the dorsal hair was removed, and the skin was disinfected with 70% ethanol. Then, 50 μL of MRSA USA 300 (~10^8^ CFU/mL) was injected subcutaneously, and after 48 h, a visible wound/abscess was observed at the inoculation site. Mice were randomly divided into five groups, and the topical treatment was initiated using the following formulations: (i) 0.5% (*w*/*w*) LyeTx I mnΔKL (2.57 µM/g of gel); (ii) 0.5% (*w*/*w*) LyeTx I mnΔK (2.73 µM/g of gel); (iii) 0.5% (*w*/*w*) mupirocin solution (9.99 µM/g of gel) (GlaxoSmithKline Brasil Ltda., Rio de Janeiro, RJ, Brazil); (iv) saline solution; or (v) formulation gel. Treatments were applied daily for three consecutive days.

Twenty-four h after the last treatment, mice were euthanized, the area around the wound/abscess was disinfected with ethanol, excised, homogenized in saline, serially diluted, and plated out on MHA supplemented with 8 μg/mL oxacillin for CFU enumeration.

### 4.17. Statistical Analyses

Statistical analyses were performed using GraphPad Prism (version 10.0, San Diego, CA, USA). All experiments were conducted in triplicate, and the results were expressed as the mean ± standard deviation (SD). Data normality was evaluated by the Shapiro–Wilk test. For normal data, One-way ANOVA with Tukey’s or Dunnett’s post hoc tests for multiple comparisons and Student’s *t*-test for pairwise comparisons were used. For non-normal data, the *t*-test or the Mann–Whitney test was used for comparisons between two groups. Area under the curve was determined for the time-kill kinetics assay. Results were considered statistically significant when *p* < 0.05.

## 5. Patents

RESENDE, J.M.; DA CRUZ NIZER, W.S.; LIMA, M.E.; DOS SANTOS, G.A.; LIMA, W.G.; SILVA, F.H.S.; VERLY, R.M.; CANDIDO, W.A.B.; ARAÚJO, G.P. “Peptídeo, composições farmacêuticas contra cepas de *Staphylococcus aureus* resistentes à meticilina, e usos”. 2026, Brazil. Patent number: BR 13 2026 012467 1, Institution: INPI—Instituto Nacional da Propriedade Industrial. Deposit: 20 May 2026.

## Figures and Tables

**Table 1:** Minimum inhibitory and bactericidal concentrations (MIC and MBC, respectively) in µM of LyeTx I mnΔKL, LyeTx I mnΔK, and vancomycin against MRSA clinical isolates.

Microorganism	LyeTx I mnΔKL		LyeTx I mnΔK		Vancomycin	
	MIC	MBC	MIC	MBC	MIC	MBC
*S. aureus* MRSA USA 300	2	2	4	4	2	16
11	4	32	16	32	2	4
29	4	>32	16	16	1	2
49	2	8	16	32	2	4
130	4	16	16	16	2	16
472	2	32	32	32	4	32
659	2	8	16	32	2	4
685	2	8	16	32	2	8
MIC_50_	2		16		2	
MBC_50_	8		32		4	

MIC_50_: Compound concentration that inhibits 50% of the tested strains; MBC_50_: Compound concentration that kills 50% of the tested strains. MIC and MBC values are expressed in µM.

**Table 2:** Fractional inhibitory concentration (FIC) index (FICI) of LyeTx I mn∆K and LyeTx I mn∆KL in combination with vancomycin against MRSA USA 300.

Peptide	FIC		FICI (ΣFIC)	Effect
	Peptide	Vancomycin		
LyeTx I mnΔKL	1	1	2	Indifferent
LyeTx I mnΔK	1	1	2	Indifferent

**Table 3:** Resensitization effect of LyeTx I mnΔKL on the antimicrobial activity of oxacillin, levofloxacin, kanamycin, and erythromycin against MRSA.

Antimicrobial Agent	MIC		
	Prior to Peptide Exposure	Post LyeTx I mnΔKL Exposure	Post LyeTx I mnΔK Exposure
Oxacillin	>128	128	64
Levofloxacin	>128	128	128
Kanamycin	>128	>128	>128
Erythromycin	>128	>128	>128

**Table 4:** Selectivity index (SI) of LyeTx I mnΔKL and LyeTx I mnΔK for MRSA against human kidney (HEK-293) and liver (Hep G2) cells.

Microorganism Identification	LyeTx I mnΔKL		LyeTx I mnΔK	
	HEK-293	HepG2	HEK-293	HepG2
*S. aureus* MRSA USA 300	2.93	2.76	8.18	7.69
11	1.46	1.38	2.05	1.92
29	1.46	1.38	2.05	1.92
49	2.93	2.76	2.05	1.92
130	1.46	1.38	2.05	1.92
472	2.93	2.76	1.02	0.96
659	2.93	2.76	2.05	1.92
685	2.93	2.76	2.05	1.92
Mean	2.76	2.60	2.61	2.46

**Table 5:** Thermodynamic parameters from the titration of POPC: POPG LUVs (20 mM) in 50 μM peptide solution at 30 °C.

Thermodynamic Parameters	LyeTx I mnΔK	LyeTx I mnΔKL
*K*_app_ (L·mol^−1^)	1.9 × 10^−4^ ± 1 × 10^−3^	3.9 × 10^−4^ ± 1 × 10^−3^
Δ*H*° (cal·mol^−1^)	− 215 ± 15	− 730 ± 20
*T*Δ*S*° (cal·mol^−1^)	5730 ± 200	5650 ± 200
Δ*S*° (cal·mol^−1^)	− 5945 ± 200	− 6380 ± 200

**Table 6:** Effect of the addition of ascorbic acid on the Minimum Inhibitory Concentration (MIC) of LyeTx I mnΔK and LyeTx I mnΔKL against MRSA USA 300.

Conditions	MIC (µM)	
	LyeTx I mnΔKL	LyeTx I mnΔK
(−) Ascorbic acid	2	4
(+) Ascorbic acid	4	32

**Table 7:** Gel formulation for in vivo studies.

Component	Concentration
Hydroxyethylcellulose (Natrosol^®^)	2.2%
Sodium metabisulfite	0.6%
Methylparaben (Nipagin^®^)	0.2%
Distilled water	-

## Data Availability

The original contributions presented in this study are included in the article/Supplementary Material. Further inquiries can be directed to the corresponding authors.

## Supplementary Materials

The following supporting information can be downloaded at https://www.mdpi.com/article/10.3390/toxins18080323/s1, Supplementary Table S1: Minimum inhibitory and bactericidal concentrations (MIC and MBC, respectively) of mupirocin against methicillin-resistant Staphylococcus aureus (MRSA). Supplementary Figure S1: MALDI-ToF mass spectra of the purified peptides: (A) LyeTx I mn∆K and (B) LyeTx I mn∆KL. The observed [M+H]^+^ ions are in agreement with the corresponding theoretical monoisotopic masses of 1828.134 Da and 1941.219 Da, respectively.

## Abbreviations

The following abbreviations are used in this manuscript:

AMP	Antimicrobial peptide
CC_50_	Cytotoxic concentration for 50% of the cells
CD	Circular dichroism
CRAB	Carbapenem-resistant *Acinetobacter baumannii*
D*_h_*	hydrodynamic diameter
DIC	1,3-diisopropylcarbodiimide
DMEM	Dulbecco’s Modified Eagle’s Medium
DMF	N,N-dimethylformamide
FBS	Fetal bovine serum
FIC	Fractional inhibitory concentration
FICI	Fractional inhibitory concentration index
HOBt	1-hydroxybenzotriazole
ITC	Isothermal titration calorimetry
LUV	Large unilamellar vesicle
MRSA	Methicillin-resistant *Staphylococcus aureus*
MBC	Minimal bactericidal concentration
MHA	Mueller–Hinton agar
MHB	Mueller–Hinton broth (MHB)
MIC	Minimal inhibitory concentration
MSA	Mannitol salt agar
MTT	3-(4,5-Dimethylthiazol-2-yl)-2,5-Diphenyltetrazolium Bromide
OD	Optical density
SI	Selective index

## References

[1] Murray C.J.L., Ikuta K.S., Sharara F., Swetschinski L., Aguilar G.R., Gray A., Han C., Bisignano C., Rao P., Wool E. (2022). Global Burden of Bacterial Antimicrobial Resistance in 2019: A Systematic Analysis. Lancet.

[2] Silva-Santana G. (2025). *Staphylococcus aureus*: Dynamics of Pathogenicity and Antimicrobial-Resistance in Hospital and Community Environments—Comprehensive Overview. Res. Microbiol..

[3] Tong S.Y.C., Davis J.S., Eichenberger E., Holland T.L., Fowler V.G. (2015). *Staphylococcus aureus* Infections: Epidemiology, Pathophysiology, Clinical Manifestations, and Management. Clin. Microbiol. Rev..

[4] Gideskog M., Melhus Å. (2019). Outbreak of Methicillin-Resistant *Staphylococcus aureus* in a Hospital Center for Children’s and Women’s Health in a Swedish County. APMIS.

[5] Bellis K.L., Dissanayake O.M., Harrison E.M., Aggarwal D. (2025). Community Methicillin-Resistant *Staphylococcus aureus* Outbreaks in Areas of Low Prevalence. Clin. Microbiol. Infect..

[6] Vestergaard M., Frees D., Ingmer H. (2019). Antibiotic Resistance and the MRSA Problem. Microbiol. Spectr..

[7] Silva V., Almeida L., Gaio V., Cerca N., Manageiro V., Caniça M., Capelo J.L., Igrejas G., Poeta P. (2021). Biofilm Formation of Multidrug-Resistant MRSA Strains Isolated from Different Types of Human Infections. Pathogens.

[8] Kaushik A., Kest H., Sood M., Steussy B.W., Thieman C., Gupta S. (2024). Biofilm Producing Methicillin-Resistant *Staphylococcus aureus* (MRSA) Infections in Humans: Clinical Implications and Management. Pathogens.

[9] CDC (2022). COVID-19: U.S. Impact on Antimicrobial Resistance.

[10] Mahjabeen F., Saha U., Mostafa M.N., Siddique F., Ahsan E., Fathma S., Tasnim A., Rahman T., Faruq R., Sakibuzzaman M. (2022). An Update on Treatment Options for Methicillin-Resistant *Staphylococcus aureus* (MRSA) Bacteremia: A Systematic Review. Cureus.

[11] Brown N.M., Goodman A.L., Horner C., Jenkins A., Brown E.M. (2021). Treatment of Methicillin-Resistant *Staphylococcus aureus* (MRSA): Updated Guidelines from the UK. JAC Antimicrob. Resist..

[12] Cong Y., Yang S., Rao X. (2019). Vancomycin Resistant *Staphylococcus aureus* Infections: A Review of Case Updating and Clinical Features. J. Adv. Res..

[13] Errecalde L., Ceriana P., Gagetti P., Erbín M., Duarte A., Rolón M.J., Cuatz D., Corso A., Kaufman S. (2013). First isolation in Argentina of community-acquired methicillin-resistant *Staphylococcus aureus* with intermediate susceptibility to vancomycin and nonsusceptibility to daptomycin. Rev. Argent. Microbiol..

[14] Hassoun A., Linden P.K., Friedman B. (2017). Incidence, Prevalence, and Management of MRSA Bacteremia across Patient Populations—A Review of Recent Developments in MRSA Management and Treatment. Crit. Care.

[15] Xuan J., Feng W., Wang J., Wang R., Zhang B., Bo L., Chen Z.-S., Yang H., Sun L. (2023). Antimicrobial Peptides for Combating Drug-Resistant Bacterial Infections. Drug Resist. Updates.

[16] Madni H., Mohamed H.A., Abdelrahman H.A.M., dos Santos-Silva C.A., Benko-Iseppon A.M., Khatir Z., Eltai N.O., Mohamed N.A., Crovella S. (2024). In Silico-Designed Antimicrobial Peptide Targeting MRSA and *E. coli* with Antibacterial and Antibiofilm Actions. Sci. Rep..

[17] Gopal R., Kim Y.G., Lee J.H., Lee S.K., Chae J.D., Son B.K., Seo C.H., Park Y. (2014). Synergistic Effects and Antibiofilm Properties of Chimeric Peptides against Multidrug-Resistant *Acinetobacter baumannii* Strains. Antimicrob. Agents Chemother..

[18] Kim W.H., Lillehoj H.S., Min W. (2017). Evaluation of the Immunomodulatory Activity of the Chicken NK-Lysin-Derived Peptide cNK-2. Sci. Rep..

[19] Duarte-Mata D.I., Salinas-Carmona M.C. (2023). Antimicrobial Peptides’ Immune Modulation Role in Intracellular Bacterial Infection. Front. Immunol..

[20] Girdhar M., Sen A., Nigam A., Oswalia J., Kumar S., Gupta R. (2024). Antimicrobial Peptide-Based Strategies to Overcome Antimicrobial Resistance. Arch. Microbiol..

[21] Santos A.T.d., Cruz G.S., Baptista G.R. (2021). Anti-Inflammatory Activities of Arthropod Peptides: A Systematic Review. J. Venom. Anim. Toxins Incl. Trop. Dis..

[22] Sabiá Júnior E.F., Menezes L.F.S., de Araújo I.F.S., Schwartz E.F. (2019). Natural Occurrence in Venomous Arthropods of Antimicrobial Peptides Active against Protozoan Parasites. Toxins.

[23] Lima W.G., Brito J.C.M., de Lima M.E., Pizarro A.C.S.T., Vianna M.A.M.d.M., de Paiva M.C., de Assis D.C.S., Cardoso V.N., Fernandes S.O.A. (2021). A Short Synthetic Peptide, Based on LyeTx I from *Lycosa erythrognatha* Venom, Shows Potential to Treat Pneumonia Caused by Carbapenem-Resistant *Acinetobacter baumannii* without Detectable Resistance. J. Antibiot..

[24] de Avelar Júnior J.T., Lima-Batista E., Castro Junior C.J., Pimenta A.M.d.C., Dos Santos R.G., Souza-Fagundes E.M., De Lima M.E. (2022). LyeTxI-b, a Synthetic Peptide Derived From a Spider Venom, Is Highly Active in Triple-Negative Breast Cancer Cells and Acts Synergistically with Cisplatin. Front. Mol. Biosci..

[25] Vieira A.P.G.C., de Souza A.N., Lima W.G., Brito J.C.M., Simião D.C., Gonçalves L.V.R., Cordeiro L.P.B., de Oliveira Scoaris D., Fernandes S.O.A., Resende J.M. (2024). The Synthetic Peptide LyeTx I mn∆K, Derived from *Lycosa erythrognatha* Spider Toxin, Is Active against Methicillin-Resistant *Staphylococcus aureus* (MRSA) In Vitro and In Vivo. Antibiotics.

[26] Santos D.M., Verly R.M., Piló-Veloso D., de Maria M., de Carvalho M.A.R., Cisalpino P.S., Soares B.M., Diniz C.G., Farias L.M., Moreira D.F.F. (2010). LyeTx I, a Potent Antimicrobial Peptide from the Venom of the Spider *Lycosa erythrognatha*. Amino Acids.

[27] Engel E., Pasini M., Kist N. (2021). Spatial Distribution and Sample Size to Estimate *Lycosa erythrognatha* (Araneae: Lycosidae) Population Density Overwintering. Braz. J. Biol..

[28] Fuscaldi L.L., de Avelar Júnior J.T., Dos Santos D.M., Boff D., de Oliveira V.L.S., Gomes K.A.G.G., Cruz R.d.C., de Oliveira P.L., Magalhães P.P., Cisalpino P.S. (2021). Shortened Derivatives from Native Antimicrobial Peptide LyeTx I: In Vitro and In Vivo Biological Activity Assessment. Exp. Biol. Med..

[29] Cardoso B.G., de Lima W.G., Fernandes S.O.A., de Lima M.E., Cardoso V.N. (2023). Antifungal Activity of a Shortened Analogue of the Natural Peptide LyeTx I Isolated from the Venom of the Spider *Lycosa erythrognatha*. Nat. Prod. Res..

[30] Radhakrishnan N., Kumar S.D., Shin S.-Y., Yang S. (2024). Enhancing Selective Antimicrobial and Antibiofilm Activities of Melittin through 6-Aminohexanoic Acid Substitution. Biomolecules.

[31] Yao A., Ma Y., Chen X., Zhou M., Xi X., Ma C., Ren S., Chen T., Shaw C., Wang L. (2021). Modification Strategy of D-Leucine Residue Addition on a Novel Peptide from *Odorrana schmackeri*, with Enhanced Bioactivity and In Vivo Efficacy. Toxins.

[32] Penesyan A., Paulsen I.T., Kjelleberg S., Gillings M.R. (2021). Three Faces of Biofilms: A Microbial Lifestyle, a Nascent Multicellular Organism, and an Incubator for Diversity. npj Biofilms Microbiomes.

[33] Liu H.Y., Prentice E.L., Webber M.A. (2024). Mechanisms of Antimicrobial Resistance in Biofilms. npj Antimicrob. Resist..

[34] Bognár B., Spohn R., Lázár V. (2024). Drug Combinations Targeting Antibiotic Resistance. npj Antimicrob. Resist..

[35] Oroojalian F., Kasra-Kermanshahi R., Azizi M., Bassami M.R. (2010). Synergistic Antibacterial Activity of the Essential Oils from Three Medicinal Plants against Some Important Food-Borne Pathogens by Microdilution Method. Iran. J. Med. Aromat. Plants Res..

[36] Matsuzaki K. (2001). Why and How Are Peptide-Lipid Interactions Utilized for Self Defence?. Biochem. Soc. Trans..

[37] Wieprecht T., Seelig J. (2002). Isothermal Titration Calorimetry for Studying Interactions between Peptides and Lipid Membranes. Current Topics in Membranes.

[38] Xia Y., Sun J., Liang D. (2014). Aggregation, Fusion, and Leakage of Liposomes Induced by Peptides. Langmuir.

[39] Smith M.C., Crist R.M., Clogston J.D., McNeil S.E. (2017). Zeta Potential: A Case Study of Cationic, Anionic, and Neutral Liposomes. Anal. Bioanal. Chem..

[40] Siddiqui A.H., Koirala J. (2025). Methicillin-Resistant *Staphylococcus aureus*. StatPearls.

[41] Zhang Q. (2025). Antimicrobial Peptides: From Discovery to Developmental Applications. Appl. Environ. Microbiol..

[42] Bucataru C., Ciobanasu C. (2024). Antimicrobial Peptides: Opportunities and Challenges in Overcoming Resistance. Microbiol. Res..

[43] César Moreira Brito J., Gustavo Lima W., Magalhães Resende J., Cristina Sampaio de Assis D., Boff D., Nascimento Cardoso V., Almeida Amaral F., Maria Souza-Fagundes E., Odília Antunes Fernandes S., Elena de Lima M. (2021). Pegylated LyeTx I-b Peptide Is Effective against Carbapenem-Resistant *Acinetobacter baumannii* in an in Vivo Model of Pneumonia and Shows Reduced Toxicity. Int. J. Pharm..

[44] Ahmad A., Yadav S.P., Asthana N., Mitra K., Srivastava S.P., Ghosh J.K. (2006). Utilization of an Amphipathic Leucine Zipper Sequence to Design Antibacterial Peptides with Simultaneous Modulation of Toxic Activity against Human Red Blood Cells. J. Biol. Chem..

[45] Wijesekara P.N.K., Kumbukgolla W.W., Jayaweera J.A.A.S., Rawat D. (2017). Review on Usage of Vancomycin in Livestock and Humans: Maintaining Its Efficacy, Prevention of Resistance and Alternative Therapy. Vet. Sci..

[46] Leiva-Sabadini C., Alvarez S., Barrera N.P., Schuh C.M., Aguayo S. (2021). Antibacterial Effect of Honey-Derived Exosomes Containing Antimicrobial Peptides Against Oral Streptococci. Int. J. Nanomed..

[47] Chen H.C., Brown J.H., Morell J.L., Huang C.M. (1988). Synthetic Magainin Analogues with Improved Antimicrobial Activity. FEBS Lett..

[48] Li Z.Q., Merrifield R.B., Boman I.A., Boman H.G. (1988). Effects on Electrophoretic Mobility and Antibacterial Spectrum of Removal of Two Residues from Synthetic Sarcotoxin IA and Addition of the Same Residues to Cecropin B. FEBS Lett..

[49] Bulet P., Hetru C., Dimarcq J.L., Hoffmann D. (1999). Antimicrobial Peptides in Insects; Structure and Function. Dev. Comp. Immunol..

[50] de Vor L., Rooijakkers S.H.M., van Strijp J.A.G. (2020). Staphylococci Evade the Innate Immune Response by Disarming Neutrophils and Forming Biofilms. FEBS Lett..

[51] da Cruz Nizer W.S., Adams M.E., Allison K.N., Montgomery M.C., Mosher H., Cassol E., Overhage J. (2024). Oxidative Stress Responses in Biofilms. Biofilm.

[52] Luo Y., Song Y. (2021). Mechanism of Antimicrobial Peptides: Antimicrobial, Anti-Inflammatory and Antibiofilm Activities. Int. J. Mol. Sci..

[53] Spoering A.L., Lewis K. (2001). Biofilms and Planktonic Cells of Pseudomonas Aeruginosa Have Similar Resistance to Killing by Antimicrobials. J. Bacteriol..

[54] Lewis K. (2001). Riddle of Biofilm Resistance. Antimicrob. Agents Chemother..

[55] Billings N., Millan M.R., Caldara M., Rusconi R., Tarasova Y., Stocker R., Ribbeck K. (2013). The Extracellular Matrix Component Psl Provides Fast-Acting Antibiotic Defense in Pseudomonas Aeruginosa Biofilms. PLoS Pathog..

[56] Colvin K.M., Gordon V.D., Murakami K., Borlee B.R., Wozniak D.J., Wong G.C.L., Parsek M.R. (2011). The Pel Polysaccharide Can Serve a Structural and Protective Role in the Biofilm Matrix of Pseudomonas Aeruginosa. PLoS Pathog..

[57] Talapko J., Meštrović T., Juzbašić M., Tomas M., Erić S., Horvat Aleksijević L., Bekić S., Schwarz D., Matić S., Neuberg M. (2022). Antimicrobial Peptides—Mechanisms of Action, Antimicrobial Effects and Clinical Applications. Antibiotics.

[58] Wimley W.C. (2010). Describing the Mechanism of Antimicrobial Peptide Action with the Interfacial Activity Model. ACS Chem. Biol..

[59] Samelo J., Mora M.J., Granero G.E., Moreno M.J. (2017). Partition of Amphiphilic Molecules to Lipid Bilayers by ITC: Low-Affinity Solutes. ACS Omega.

[60] Wadhwani P., Reichert J., Bürck J., Ulrich A.S. (2012). Antimicrobial and Cell-Penetrating Peptides Induce Lipid Vesicle Fusion by Folding and Aggregation. Eur. Biophys. J..

[61] Goswami M., Mangoli S.H., Jawali N. (2006). Involvement of Reactive Oxygen Species in the Action of Ciprofloxacin against *Escherichia coli*. Antimicrob. Agents Chemother..

[62] Kohanski M.A., Dwyer D.J., Hayete B., Lawrence C.A., Collins J.J. (2007). A Common Mechanism of Cellular Death Induced by Bactericidal Antibiotics. Cell.

[63] Wang X., Zhao X. (2009). Contribution of Oxidative Damage to Antimicrobial Lethality. Antimicrob. Agents Chemother..

[64] da Cruz Nizer W.S., Inkovskiy V., Versey Z., Strempel N., Cassol E., Overhage J. (2021). Oxidative Stress Response in Pseudomonas Aeruginosa. Pathogens.

[65] Lin L., Chi J., Yan Y., Luo R., Feng X., Zheng Y., Xian D., Li X., Quan G., Liu D. (2021). Membrane-Disruptive Peptides/Peptidomimetics-Based Therapeutics: Promising Systems to Combat Bacteria and Cancer in the Drug-Resistant Era. Acta Pharm. Sin. B.

[66] Liu Q., He D., Wang L., Wu Y., Liu X., Yang Y., Chen Z., Dong Z., Luo Y., Song Y. (2024). Efficacy and Safety of Antibiotics in the Treatment of Methicillin-Resistant *Staphylococcus aureus* (MRSA) Infections: A Systematic Review and Network Meta-Analysis. Antibiotics.

[67] Brazilian Committee on Antimicrobial Susceptibility Testing (BrCAST) (2017). Orientações do EUCAST para a Detecção de Mecanismos de Resistência e Resistências Específicas de Importância Clínica e/ou Epidemiológica.

[68] Chan W.C., White P.D. (2000). Fmoc Solid Phase Peptide Synthesis: A Practical Approach.

[69] Troll W., Cannan R.K. (1953). A Modified Photometric Ninhydrin Method for the Analysis of Amino and Imino Acids. J. Biol. Chem..

[70] (2024). Methods for Dilution Antimicrobial Susceptibility Tests for Bacteria That Grow Aerobically.

[71] Herrera K.M.S., da Silva F.K., de Lima W.G., Barbosa C.d.S., Gonçalves A.M.M.N., Viana G.H.R., Soares A.C., Ferreira J.M.S. (2020). Antibacterial and Antibiofilm Activities of Synthetic Analogs of 3-Alkylpyridine Marine Alkaloids. Med. Chem. Res..

[72] Stindlova M., Peroutka V., Jencova V., Havlickova K., Lencova S. (2024). Application of MTT Assay for Probing Metabolic Activity in Bacterial Biofilm-Forming Cells on Nanofibrous Materials. J. Microbiol. Methods.

[73] Twentyman P., Luscombe M. (1987). A Study of Some Variables in a Tetrazolium Dye (MTT) Based Assay for Cell Growth and Chemosensitivity. Exp. Oncol..

[74] Orhan G., Bayram A., Zer Y., Balci I. (2005). Synergy Tests by E Test and Checkerboard Methods of Antimicrobial Combinations against Brucella Melitensis. J. Clin. Microbiol..

[75] Gęgotek A., Skrzydlewska E. (2023). Ascorbic Acid as Antioxidant. Vitam. Horm..

[76] Kielkopf C.L., Bauer W., Urbatsch I.L. (2020). Bradford Assay for Determining Protein Concentration. Cold Spring Harb. Protoc..

[77] Kirby C., Gregoriadis G. (1984). Dehydration-Rehydration Vesicles: A Simple Method for High Yield Drug Entrapment in Liposomes. Nat. Biotechnol..

[78] ANVISA—Agencia Nacional de Vigilância Sanitária (2019). Farmacopéia Brasileira.

[79] Lima W.G., de Brito J.C.M., Cardoso V.N., Fernandes S.O.A. (2021). In-Depth Characterization of Antibacterial Activity of Melittin against *Staphylococcus aureus* and Use in a Model of Non-Surgical MRSA-Infected Skin Wounds. Eur. J. Pharm. Sci..

[80] Thangamani S., Mohammad H., Abushahba M.F.N., Sobreira T.J.P., Seleem M.N. (2016). Repurposing Auranofin for the Treatment of Cutaneous Staphylococcal Infections. Int. J. Antimicrob. Agents.

