# Supplementary material for: LyeTx I mnΔKL, a New Synthetic Peptide Derived from a Lycosa erythrognatha Toxin, with Potent In Vitro and In Vivo Activity Against Methicillin-Resistant Staphylococcus aureus
Source: Toxins (Basel). 2026 Jul 25;18(8):323. doi: 10.3390/toxins18080323 (PMC13517607; doi:10.3390/toxins18080323)
Supplement: Supplementary file 1 [file toxins-18-00323-s001.zip › toxins-4429944-supplementary.pdf]

Supplementary Materials

Supplementary Table S1. Minimum inhibitory and bactericidal concentrations (MIC and MBC, respectively) of mupirocin against methicillin-resistant *Staphylococcus aureus* (MRSA).

**Microorganism identification**	**MIC (µg/mL)**	**MBC (µg/mL)**
S. aureus MRSA USA 300	0.125	> 0.125
11	0.0625	> 0.125
29	0.125	> 0.125
49	0.125	> 0.125
130	0.0625	> 0.125
472	0.0625	> 0.125
659	0.125	> 0.125
685	0.0625	> 0.125

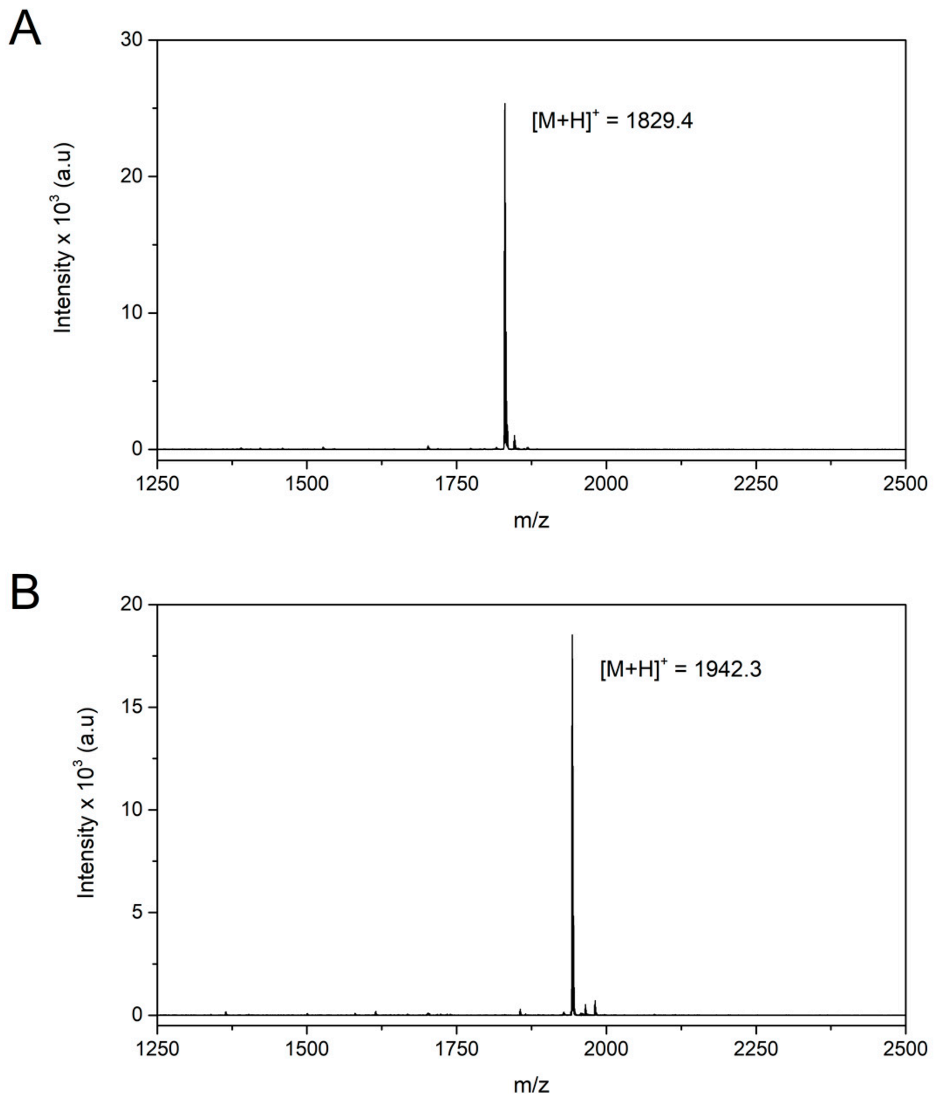

Supplementary Figure S1. MALDI-ToF mass spectra of the purified peptides: (A) LyeTx I mnΔK and (B) LyeTx I mnΔKL. The observed [M+H]$^+$ ions are in agreement with the corresponding theoretical monoisotopic masses of 1828.134 Da and 1941.219 Da, respectively.
